# Association of plasma ceramide with decline in kidney function in patients with type 2 diabetes

**DOI:** 10.1016/j.jlr.2024.100552

**Published:** 2024-05-03

**Authors:** Resham L. Gurung, Yiamunaa M, Wai Kin Tham, Sylvia Liu, Huili Zheng, Janus Lee, Keven Ang, Markus Wenk, Tavintharan Subramaniam, Chee Fang Sum, Federico Torta, Jian-Jun Liu, Su Chi Lim

**Affiliations:** 1 Clinical Research Unit, Khoo Teck Puat Hospital, Singapore, Singapore; 2 Cardiovascular and Metabolic Disorders, Duke-NUS Medical School, Singapore, Singapore; 3 Precision Medicine Translational Research Programme and Department of Biochemistry, Yong Loo Lin School of Medicine, National University of Singapore, Singapore, Singapore; 4 SLING, Singapore Lipidomics Incubator, Life Sciences Institute, National University of Singapore, Singapore, Singapore; 5 Diabetes Centre, Admiralty Medical Centre, Singapore, Singapore; 6 Saw Swee Hock School of Public Health, Singapore, Singapore; 7 Lee Kong Chian School of Medicine, Nanyang Technological University, Singapore, Singapore

**Keywords:** diabetes kidney disease, ceramide ratio, ceramide score, end-stage kidney disease

## Abstract

Circulating ceramide levels are dysregulated in kidney disease. However, their associations with rapid decline in kidney function (RDKF) and end-stage kidney disease (ESKD) in patients with type 2 diabetes (T2D) are unknown. In this prospective study of 1746 T2D participants, we examined the association of plasma ceramide Cer16:0, Cer18:0, Cer24:0, and Cer24:1 with RDKF, defined as an estimated glomerular filtration rate (eGFR) decline of 5 ml/min/1.73 m^2^ per year or greater, and ESKD defined as eGFR <15/min/1.73 m^2^ for at least 3 months, on dialysis or renal death at follow-up. During a median follow-up period of 7.7 years, 197 patients experienced RDKF. Ceramide Cer24:0 (odds ratio [OR] = 0.71, 95% CI 0.56–0.90) and ratios Cer16:0/Cer24:0 (OR = 3.54 [1.70–7.35]), Cer18:0/Cer24:0 (OR = 1.89 [1.10–3.25]), and Cer24:1/Cer24:0 (OR = 4.01 [1.93–8.31]) significantly associated with RDKF in multivariable analysis; 124 patients developed ESKD. The ratios Cer16:0/Cer24:0 (hazard ratio [HR] = 3.10 [1.44–6.64]) and Cer24:1/Cer24:0 (HR = 4.66 [1.93–11.24]) significantly associated with a higher risk of ESKD. The Cer24:1/Cer24:0 ratio improved risk discrimination for ESKD beyond traditional risk factors by small but statistically significant margin (Harrell C-index difference: 0.01; *P* = 0.022). A high ceramide risk score also associated with RDKF (OR = 2.28 [1.26–4.13]) compared to lower risk score. In conclusion, specific ceramide levels and their ratios are associated with RDKF and conferred an increased risk of ESKD, independently of traditional risk factors, including baseline renal functions in patients with T2D.

Diabetic kidney disease (DKD) is the leading cause of end-stage kidney disease (ESKD), cardiovascular events, and reduced life span among patients with type 2 diabetes (T2D) (1, 2, 3, 4). Although several antiglycemic, antihypertension, and renal protective agents are available to reduce the risk of developing DKD and ESKD, considerable residual risk remains (5). The rapid decline in kidney function (RDKF) is a common pathway leading to ESKD and an independent risk factor for cardiovascular events and mortality in patients with T2D (6, 7, 8). However, the decline in kidney function is heterogenous, beyond that captured by traditional cardio-renal risk factors such as obesity, hypertension, dyslipidemia, and smoking (9). Hence, discovering novel biomarkers is essential to aid the stratification of T2D patients for early intervention to prevent or slow down RDKF.

Ceramides are biologically active sphingolipids comprising a long-chain base and a fatty acyl chain, differing in chain length (C16–C26) and degree of unsaturation (10). Ceramides are synthesized by six different ceramide synthases (CERS1-CERS6), each having unique tissue distributions and substrate specificities (11) and are involved in pathways related to cellular damage signaling, inflammation, and apoptosis (12, 13, 14, 15). Circulating plasma ceramides such as Cer(d18:1/16:0), Cer(d18:1/18:0), Cer(d18:1/24:0), and Cer(d18:1/24:1) (hereby referred to as Cer16:0, Cer18:0, Cer24:0, and Cer24:1, respectively, for simplicity) are associated with adverse cardiovascular events, independent of traditional risk factors (16, 17, 18, 19). Accumulating evidence from animal models and clinical studies suggest that ceramide levels are also dysregulated in DKD (20). For example, *Sas et al.* showed that the ceramide level in kidney cortices decreased with more advanced DKD in mouse models (21). We and others have shown that ceramide levels are significantly altered in patients with chronic kidney disease (CKD) compared to those without CKD (22, 23, 24).

Recent studies have demonstrated the potential of ceramide ratios to estimate the risk for cardiovascular disease. For instance, ratio to Cer 24:0 may be of a greater prognostic value for cardiovascular events than the concentration of individual ceramides (16, 19, 25, 26). In addition, studies have also demonstrated the improved predictive value of the ceramide-based risk score to identify patients at greater risk of cardiovascular events, when compared to low density lipoprotein (LDL) cholesterol (17, 18, 27, 28). However, less is known about the role of ceramides in patients with DKD. Given that the risk factors and molecular mechanisms in diabetes and cardiovascular events are closely linked with CKD, we hypothesized that plasma ceramide levels might be associated with a declining kidney function among T2D (29). Therefore, we used a large T2D prospective cohort to evaluate the association of ceramide, ceramide ratios, and risk score with RDKF and ESKD, independent of baseline traditional cardio-renal risk factors.

## Materials and Methods

### Study population and design

The Singapore Study of Macro-Angiopathy and Micro-Vascular Reactivity in Type 2 Diabetes (SMART2D) cohort, as described previously (30, 31), is an ongoing prospective cohort study with 2052 T2D patients of Southeast Asian ancestry (Chinese, Malay, Indian, and others), aged 21–90 years at baseline, recruited between 2011 and 2014. Baseline demographic and clinical data and biological samples were collected at the time of enrolment. Participants were actively followed up every 3 years, and clinical data were also reviewed from their electronic medical records in a centralized database. The date of the last clinical visit was censored on December 31, 2021. We excluded 43 T2D patients with eGFR < 15 ml/min/1.73 m^2^ at baseline and 106 participants with no consent for future research or no blood samples. A total of 1,903 participants had baseline ceramide measured. We excluded 157 participants with eGFR< 30 and no follow-up eGFR data. Finally, 1,746 participants were included for analysis (Supplemental Fig. S1). All participants gave informed consent, and the current study was approved by the Singapore National Health Group Domain Specific Review Board and conducted in compliance with the Declaration of Helsinki principles.

### Clinical outcomes

The primary outcome was annual eGFR decline estimated by mixed linear model. Participants with at least three eGFR readings were included in this analysis. We defined RDKF as an eGFR decline of 5 ml/min/1.73 m^2^ per year or more, following the Kidney Disease: Improving Global Outcomes guidelines (32, 33). The secondary outcome was incident ESKD defined as follows: *1)* follow-up eGFR < 15 ml/min/1.73 m^2^ per year for at least 3 months; *2)* sustained dialysis for >3 months; or *3)* death related to renal causes, whichever occur first. Deaths were identified from electronic medical records and by linkage with the national death registry. Renal death was identified based on primary diagnosis on the death certificate.

### Plasma ceramides measurement

Ceramides in human plasma samples were extracted using a 1:1 (v/v) butanol/methanol mixture. Ten microliters of plasma were thawed at 4°C mixed with 490 μL of butanol/methanol containing the internal standards [D7-Cer d18:1/16:0 (0.0025 μM), D7-Cer d18:1/18:0 (0.001 μM), D7-Cer d18:1/24:0 (0.03 μM), and D7-Cer d18:1/24:1 (0.01 μM) purchased from Avanti Polar Lipids]. The mixture was shaken for 1 h at 1,500 rpm with an orbital shaker and centrifuged at 3,500 rcf at 10°C for 20 min. The extract was then injected via RapidFire (Agilent Technologies) into a 6495C Agilent triple quadrupole mass spectrometer (QQQ) for analysis.

A RapidFire 365 solid-phase extraction (SPE) (Agilent Technologies, Santa Clara, CA) was interfaced to an Agilent 6495C QQQ MS system (Agilent Technologies, Santa Clara, CA). The RapidFire works as an ultrafast online SPE. The use of a C18 SPE cartridge (Agilent Technologies, Santa Clara, CA) was optimized for ceramides binding using pure standards and commercial plasma. Agilent Rapidfire injections were conducted at room temperature, and samples were aspirated into the sample loop at 1.5 ml/min for 600 ms and loaded into an in-line C18, 12 ml SPE cartridge using a loading buffer made of 60:40 (v/v) water:acetonitrile + 10 mM ammonium formate at a flow rate of 1.5 ml/min for 3,000 ms, followed by elution with 10:90 (v/v) acetonitrile:isopropanol + 10 mM ammonium formate at a flow rate of 0.4 ml/min for 10,000 ms. The cartridge was then re-equilibrated with the loading buffer at 1.5 ml/min for 500 ms. Total analysis time was approximately 15 s per sample.

Mass spectrometry was performed using the Agilent 6495C triple quadrupole system with electrospray ionization in positive ion multiple reaction monitoring mode with an ion-spray voltage of 3,500 kV and a desolvation temperature of 200°C. The multiple reaction monitoring transitions monitored for each endogenous ceramide were as follows: Cer 16:0 (538.5 → 264.3), Cer 18:0 (566.6 → 264.3), Cer 24:0 (650.6 → 264.3), and Cer 24:1 (648.6 → 264.3). The transitions monitored for the deuterated ceramides were as follows: D7 Cer 16:0 (545.6 → 271.3), D7 Cer 18:0 (573.6 → 271.3), D7 Cer 24:0 (657.7 → 271.3), and D7 Cer 24:1 (655.7 → 271.3). Collision energy 30 V was used for all deuterated and nondeuterated ceramide transitions.

Raw data were analyzed using Agilent MassHunter QQQ Quant 10.1 software, and isotopic corrections for Cer 24:0 and D7 Cer 24:0 were done using LICAR software, as reported in Gao *et al.* (2021) (34). Concentrations (conc.) of each ceramide were calculated using a one-point calibration, in a linear interval, by the formula

$$Conc.\;of\;analyte=\frac{Area\;of\;Endogenous}{Area\;of\;InternalStandard}\times \frac{Volume\;ofInternalStandard}{Sample\;amount}\times Conc.\;of\;IS$$

The concentration of ceramide are expressed in μM. For quality assurance/quality control measures, we used commercial long term reference plasma to account for extraction variation, SRM 1950 plasma (National Institute of Standards and Technology reference plasma) for an additional batch correction sample, and technical quality control (pooled extract) which is used to track system response drift. The coefficient of variation between plates was 6.71%–10.2%. All samples had individually measured concentrations (no missing values).

### Clinical and biochemical variables

Baseline information on sociodemographic, medical history, and medication usage was obtained by experienced research nurses using a questionnaire. Ethnicity and smoking status were self-reported. Body mass index (BMI) was calculated by weight in kilograms divided by the square of height in meters. Blood pressure was measured using an automated blood pressure monitor, and an average of three readings was used. Glycated hemoglobin (HbA1c) was measured by immunoturbidimetric method (COBAS Integra 800 analyzer; Roche, Basel, Switzerland). Triglyceride, high density lipoprotein (HDL), and LDL cholesterol were quantified by enzymatic methods (cobas c system; Roche Diagnostic GmbH, Mannheim, Germany). Urinary albumin was measured by solid-phase competitive chemiluminescent enzymatic immunoassay (Immulite, DPC, Gwynedd, UK). Albuminuria was expressed as an albumin-to-creatinine ratio (ACR, mg/g). The eGFR was calculated with the Chronic Kidney Disease Epidemiology Collaboration formula (35).

### Statistical analysis

Baseline continuous clinical and biochemical variables were presented as mean with standard deviation (SD) for normally distributed variables and median (interquartile range, IQR) for skewed variables. Categorical variables were presented as proportions. Between-group differences were compared by independent *t* test for normally distributed variables or Mann-Whitney U test for nonnormally distributed variables. The chi^2^ test was used for categorical variables. Ceramides concentrations in plasma were considered as follows: *1)* individually (per SD); *2)* as a ratio to Cer24:0 (natural log-transformed); and *3)* as ceramide risk score (CERT). The ceramide score was calculated based on levels of Cer16:0, Cer18:0, and Cer24:1 and their corresponding ratio with Cer24:0 as described previously (16, 19). Briefly, each ceramide variable (six in total) were compared among the whole study population, including those without follow-up eGFR data, and risk points were given based on which quartile the variable belonged to. For example, variable in quartiles 1 and 2 were assigned with a 0 score, while those in quartiles 3 and 4 were assigned 1 and 2, respectively. This was repeated for all six ceramide variables (three individual ceramides and three ratios). Based on the accumulated score, participants were grouped into four risk categories: lower (0–2), moderate (3, 4, 5, 6), increased (7,8,9), and high risk (10, 11, 12).

The eGFR slope has been proposed as a surrogate for kidney outcomes in clinical trials [31, 32, 35]. In this study, we used a linear mix model to generate the eGFR slope (random intercept, random slope, and time coefficient) using pooled eGFR readings from research and clinical visits after excluding those measured during hospitalization. We used multivariable logistic regression and Cox regression models to assess the odds ratios (ORs) for RDKF and hazard ratio (HR) for ESKD, respectively. Model 1 included age, sex, ethnicity, BMI, HbA1c, diabetes duration, mean arterial pressure (MAP), LDL-cholesterol, triglyceride (natural log-transformed), cardiovascular disease history, and smoking status; Model 2 included variables in Model 1 and baseline eGFR, urine ACR (uACR) (natural log-transformed), and renin-angiotensin system (RAS) antagonist. The proportional hazard assumption was assessed by Schoenfeld residuals.

We also performed sensitivity analysis. We stratified participants by CKD status (eGFR <60 vs. ≥60 ml/min/1.73 m^2^) and albuminuria category (uACR <30 mg/g, 30–299 mg/g, and ≥300 mg/g) to examine the association between ceramide and RDKF and ESKD in subgroups. In the exploratory analysis, we evaluated whether ceramide improved the prediction of RDKF and ESKD above the traditional cardiometabolic risk factors using the Harrell’s C-statistic (36).

Data analysis was performed using SPSS (version 27) and R (version 386.4.0.0). A 2-sided *P* value < 0.05 was considered statistically significant.

## Results

### Elevated ceramide levels are independently associated with RDKF

During a median follow-up period of 7.7 (IQR 4.7–8.9) years, 197 participants (11%) experienced RDKF. Compared to participants without RDKF, participants with RDKF had a longer duration of diabetes, higher HbA1c, higher blood pressure, higher total cholesterol and triacylglycerol, and poorer renal function (lower eGFR, higher uACR) and were more likely to be a smoker and on RAS antagonist and insulin (Table 1). The baseline plasma concentration of long-chain ceramide (Cer16:0, Cer18:0) and very long-chain ceramide (Cer24:1), ratios Cer16:0/Cer24:0, Cer24:1/Cer24:0, and the proportion of participants with high-risk score (15.7% vs. 8.5%) were significantly higher among participants with RDKF than those without RDKF (Table 2). All four measured ceramides significantly correlated with HbA1c, diabetes duration, total cholesterol, LDL-cholesterol and triacylglycerols, diastolic blood pressure, and uACR (Supplemental Fig. S2).

**Table 1:** Baseline clinical and biochemical characteristics stratified by RDKF status at follow-up

	All participants	Non-RDKF	RDKF	*P*
	(N = 1746)	(N = 1549)	(N = 197)	
Index age (years)	57.2 ± 10.7	57.3 ± 10.7	56.1 ± 10.7	0.138
Male sex (%)	50.4	50.4	50.8	0.914
**Ethnicity (%)**				**<0.001**
Chinese	51.9	52.9	43.7	
Malay	21	18.4	41.1	
Asian Indian	27.1	28.7	15.2	
**Diabetes duration (years)**	**11 ± 8.9**	**10.8 ± 8.7**	**13.3 ± 9.7**	**<0.001**
**Active smoker (%)**	**8.9**	**8.4**	**12.7**	**0.047**
ASCVD history (%)	7.9	7.8	8.6	0.690
Body mass index (kg/m^2^)	27.7 ± 5.3	27.7 ± 5.2	28.2 ± 5.8	0.219
**HbA1c (%)**	**7.8 ± 1.3**	**7.69 ± 1.26**	**8.42 ± 1.58**	**<0.001**
**Blood pressure (mmHg)**				
** Systolic**	**140 ± 18**	**139 ± 18**	**149 ± 20**	**<0.001**
** Diastolic**	**79 ± 9**	**79 ± 9**	**82 ± 9**	**<0.001**
** Mean arterial pressure**	**99.0 ± 11.0**	**98.8 ± 10.4**	**104.1 ± 10.9**	**<0.001**
Lipid profile (mM)				
** Total cholesterol**	**4.40 ± 0.99**	**4.38 ± 0.90**	**4.62 ± 1.54**	**0.028**
HDL cholesterol	1.30 ± 0.36	1.31 ± 0.36	1.26 ± 0.35	0.172
LDL cholesterol	2.74 ± 0.81	2.72 ± 0.79	2.82 ± 0.91	0.081
** Triacylglycerol (IQR)**	**1.38 (1.03–1.91)**	**1.37 (1.03–1.89)**	**1.51 (1.10–2.16)**	**0.004**
Baseline renal function				
** eGFR (ml/min/1.73 m^2^)**	**86.0 ± 23.0**	**89.0 ± 23.0**	**85.3 ± 23.7**	**0.037**
** urine ACR (μg/mg, IQR)**	**21 (7–88)**	**18 (6–62)**	**231 (32–1089)**	**<0.001**
Medication usage (%)				
** RAS antagonist**	**60.2**	**58.4**	**78.6**	**<0.001**
** ACE inhibitor**	**37.4**	**36.0**	**48.5**	**0.001**
** ARB**	**26.2**	**24.7**	**37.2**	**<0.001**
** Insulin**	**27.4**	**25.3**	**44.6**	**<0.001**
Statin	80.6	80.3	83.1	0.357

Data were presented as mean ± SD, median (interquartile range, IQR), or percentages. Between-group differences were compared by Student’s *t*-test, Mann-Whitney U test or chi^2^ test where appropriate. Variables differed significantly between groups have been highlighted in bold font. RAS antagonist represents patients on at least one of either ACE inhibitor or ARB.

ACE, angiotensin converting enzyme; ACR, albumin-to-creatinine ratio; ARB, angiotensin receptor blockers; ASCVD, atherosclerotic cardiovascular disease; eGFR, estimated glomerular filtration function; RAS, renin-angiotensin system; RDKF, rapid decline in kidney function.

**Table 2:** Baseline plasma ceramide levels stratified by RDKF status at follow-up

	All Participants	Non-RDKF	RDKF	*P*
Individual ceramide				
**Cer16:0**	**0.236 (0.200–0.279)**	**0.233 (0.198–0.276)**	**0.250 (0.213–0.302)**	**0.001**
**Cer18:0**	**0.107 (0.084–0.134)**	**0.106 (0.083–0.134)**	**0.115 (0.091–0.141)**	**0.029**
Cer24:0	3.500 (2.887–4.198)	3.496 (2.888–4.180)	3.541 (2.865–4.386)	0.264
**Cer24:1**	**1.044 (0.853–1.264)**	**1.033 (0.851–1.242)**	**1.166 (0.893–1.400)**	**<0.001**
Ratios with Cer24:0				
**Cer16:0/Cer24:0**	**0.067 (0.059–0.078)**	**0.067 (0.058–0.078)**	**0.070 (0.060–0.084)**	**<0.001**
Cer18:0/Cer24:0	0.031 (0.025–0.038)	0.031 (0.025–0.038)	0.032 (0.025–0.040)	0.063
**Cer24:1/Cer24:0**	**0.302 (0.256–0.351)**	**0.300 (0.255–0.349)**	**0.318 (0.267–0.374)**	**<0.001**
Ceramide risk score				**<0.001**
Lower risk (0–2)	593 (34.0)	547 (35.3)	46 (23.4)	
Moderate risk (3–6)	670 (38.4)	592 (38.2)	78 (39.6)	
Increased risk (7–9)	320 (18.3)	278 (17.9)	42 (21.3)	
High risk (10–12)	163 (9.3)	132 (8.5)	31 (15.7)	

Data are presented as median [IQR] for individual ceramide (μM) and ratios and proportion (n,%) for ceramide risk score. Variables differed significantly between groups have been highlighted in bold. Ceramide risk score was based on levels of Cer16:0,Cer18:0,Cer24:1 and their corresponding ratio with Cer24:0. Briefly, each ceramide variable (six in total) were compared among the whole study population, and risk points were given based on which quartile the variable belonged to. Variable in quartiles 1 and 2 were assigned with a 0 score, while those in quartiles 3 and 4 were assigned 1 and 2, respectively. Based on the accumulated score, participants were grouped into four risk categories: lower (0–2), moderate (3–6), increased (7–9), and high risk (10–12).

Cer, ceramide; RDKF, rapid decline in kidney function.

**Fig. 1:**
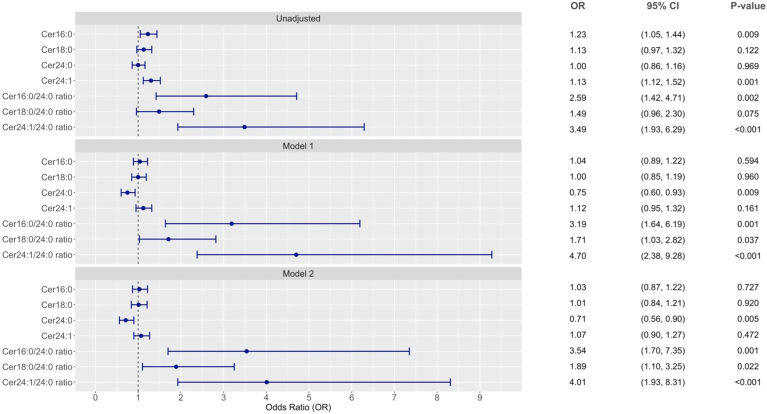
Forest plot of associations of individual ceramide and ceramide ratio with RDKF. Odds ratio are per SD for individual ceramide or per natural log transformed for ceramide ratio. Model 1: adjusted age, sex, ethnicity (Chinese as reference), CVD history (no as reference), smoking status (current smoker vs. others), body mass index, diabetes duration, HbA1c, mean arterial pressure, LDL-cholesterol, and triglyceride. Model 2: additionally adjusted for baseline eGFR and urine ACR (log-transformed) and RAS antagonist usage above model 1.

Figure 1 shows the association of the ceramide levels with RDKF. Among the four individual ceramide species, only Cer24:0 (OR = 0.71, 95% CI 0.56–0.90, *P* = 0.005) was significantly associated with RDKF after adjusting for the cardiometabolic risk factors and baseline renal function (Supplemental Table S1). The three ceramide ratios, Cer16:0/Cer24:0, Cer18:0/Cer24:0, and Cer24:1/Cer24:0, were significantly associated with RDKF independently of the cardiometabolic risk factors and baseline renal function with ORs of 3.54 (95% CI 1.70–7.35, *P* = 0.001), 1.89 (95% CI 1.10–3.25, *P* = 0.022), and 4.01 (95% CI 1.93–8.31, *P* < 0.001), respectively (Supplemental Table S2).

**Fig. 2:**
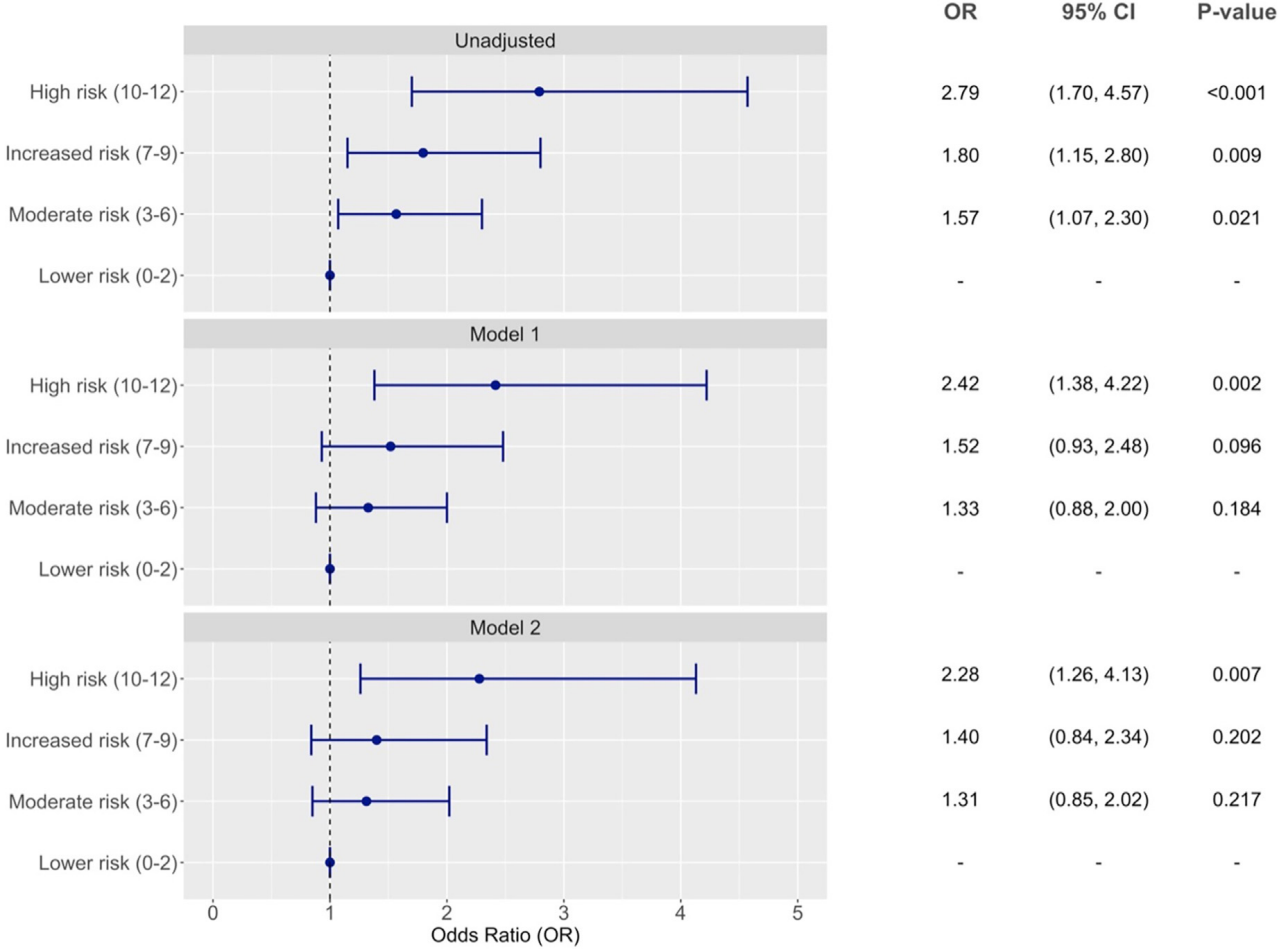
Forest plot of association of ceramide score with RDKF. Model 1: adjusted age, sex, ethnicity (Chinese as reference), CVD history (no as reference), smoking status (current smoker vs. others), body mass index, diabetes duration, HbA1c, mean arterial pressure, LDL-cholesterol, and triglyceride. Model 2: additionally adjusted for baseline eGFR and urine ACR (log-transformed) and RAS antagonist usage above model 1. Low score (0–2) was used as reference group.

Figure 2 shows the association of the CERT with RDKF. In unadjusted analysis, moderate risk (OR = 1.57, 95% CI 1.07–2.30, *P* = 0.021), increased risk (OR = 1.80, 95% CI 1.15–2.80, *P* = 0.009), and high risk (OR = 2.79, 95% CI 1.70–4.57, *P* < 0.001) scores were significantly associated with increased ORs for RDKF compared to the lower risk score (Supplemental Table S3). However, only high-risk score remained significantly associated with RDKF after adjusting for cardiometabolic risk factors and baseline renal functions (OR = 2.28, 95% CI 1.26–4.13, *P* = 0.007).

### Ceramide ratios predict risk of ESKD

During a median follow-up period of 7.7 years, 124 participants (7%) developed ESKD. Compared to participants without ESKD, participants with ESKD had a longer duration of diabetes, higher HbA1c, higher blood pressure, lower HDL cholesterol, higher triacylglycerol, and poorer renal function (lower eGFR and higher uACR) and were more likely to be a Malay ethnicity, on RAS antagonist and insulin (Supplemental Table S4). The baseline plasma concentration of Cer16:0 and Cer24:1, ratios Cer16:0/Cer24:0, Cer24:1/Cer24:0, and the proportion of participants in increased risk and high-risk score were significantly higher among participants who developed ESKD than those without ESKD (Supplemental Table S5).

**Fig. 3:**
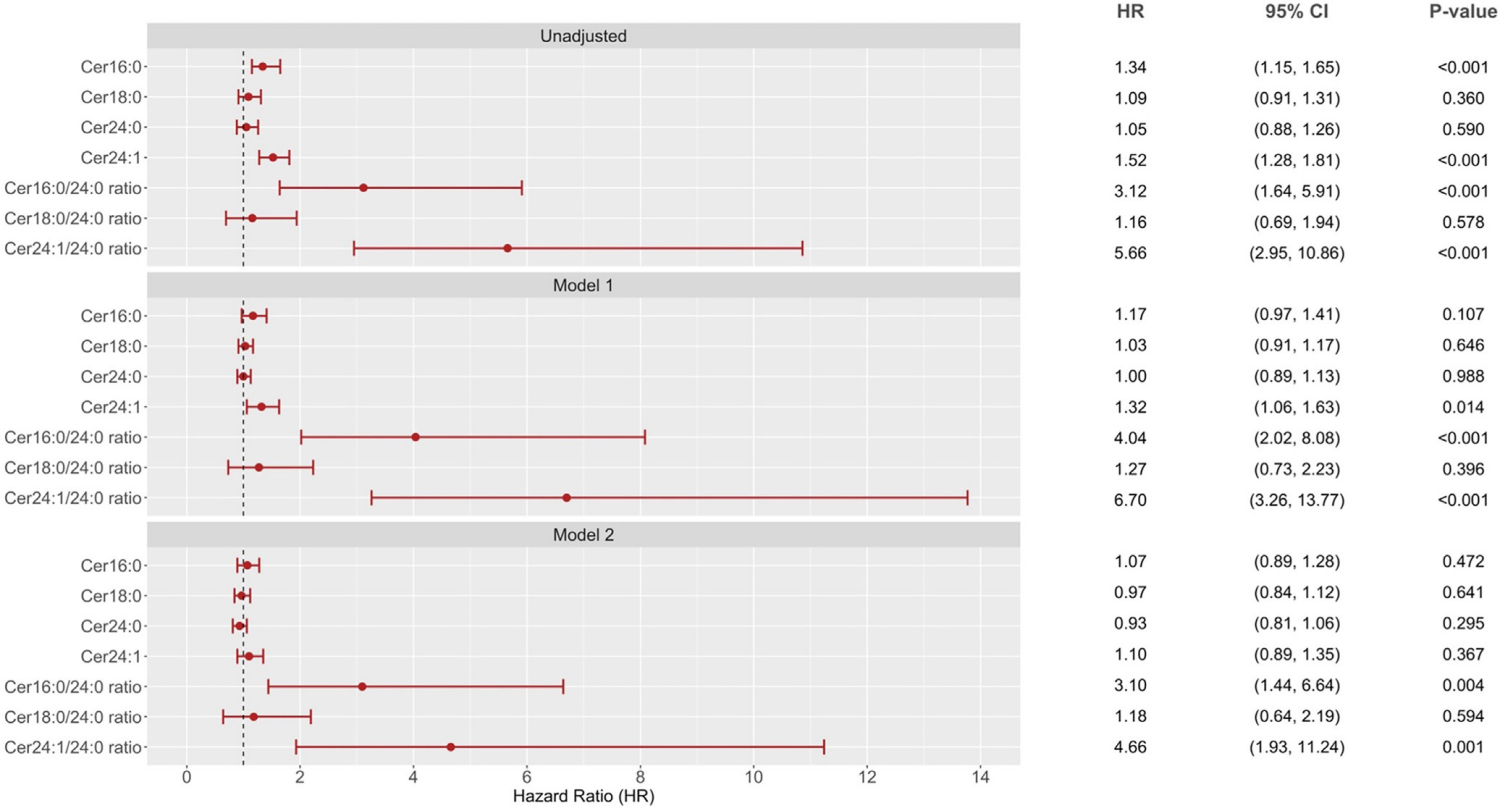
Forest plot of associations of individual ceramide and ceramide ratios with ESKD. Hazard ratio is per SD for individual ceramide or per natural log transformed for ceramide ratio. Model 1: adjusted age, sex, ethnicity (Chinese as reference), CVD history (no as reference), smoking status (current smoker vs. others), body mass index, diabetes duration, HbA1c, mean arterial pressure, LDL-cholesterol, and triglyceride. Model 2: additionally adjusted for baseline eGFR and urine ACR (log-transformed) and RAS antagonist usage above model 1.

Figure 3 shows the associations of the ceramides with incident ESKD. In unadjusted analysis, elevated levels of Cer16:0 (HR = 1.34, 95% CI 1.15–1.65, *P* < 0.001) and Cer24:1 (HR = 1.52, 95% CI 1.28–1.81, *P* < 0.001) were significantly associated with increased risk of ESKD. However, only Cer24:1 (HR = 1.32, 95% CI 1.06–1.63, *P* = 0.014) remained significantly associated after adjusting for cardio-renal risk factors. The association was attenuated after further adjustment for baseline renal functions (HR = 1.10. 95% CI 0.89–1.35, *P* = 0.367). In unadjusted analysis, the ratios Cer16:0/Cer24:0, Cer18:0/Cer24:0, and Cer24:1/Cer24:0 were significantly associated with increased risk of ESKD with HRs of 3.12 (95% CI 1.64–5.91, *P* < 0.001), 1.16 (95% CI 0.69–1.94, *P* = 0.578), and 5.66 (95% CI 2.95–10.86, *P* = <0.001), respectively. The ratios Cer16:0/Cer24:0 and Cer24:1/Cer24:0 remained significantly associated with increased risk of ESKD after adjustment for cardiometabolic risk factors, including baseline renal functions (Fig. 3) (Supplemental Table S6).

**Fig. 4:**
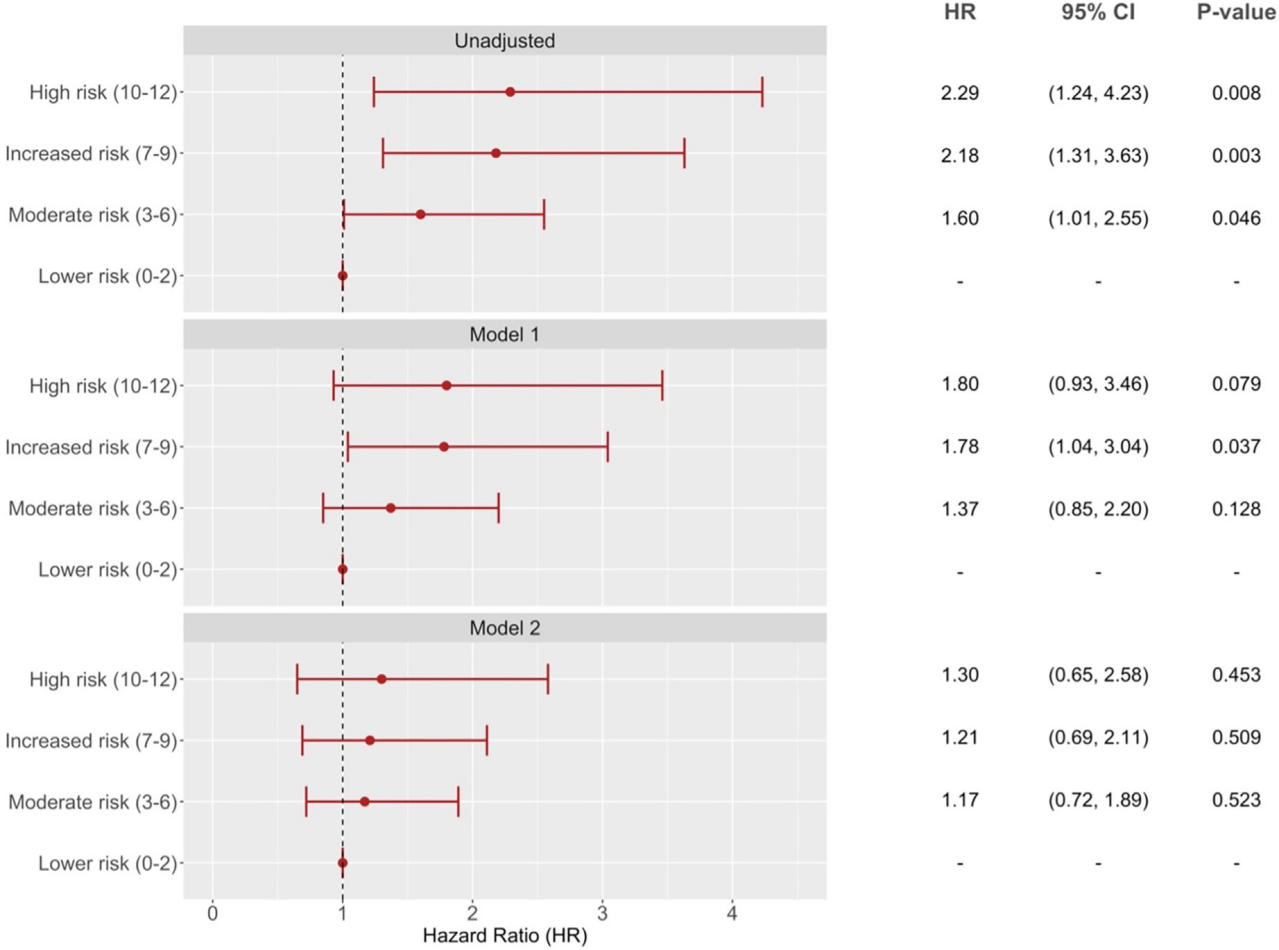
Forest plot of association of ceramide score with ESKD. Model 1: adjusted age, sex, ethnicity (Chinese as reference), CVD history (no as reference), smoking status (current smoker vs. others), body mass index, diabetes duration, HbA1c, and mean arterial pressure. Model 2: additionally adjusted for baseline eGFR and urine ACR (log-transformed) and RAS antagonist usage above model 1. Low score (0–2) was used as reference group.

Figure 4 shows the association of the CERT with ESKD. In unadjusted analysis, moderate risk (HR = 1.60, 95% CI 1.01–2.55, *P* = 0.046), increased risk (HR = 2.18, 95% CI 1.31–3.63, *P* = 0.003), and high-risk (HR = 2.29, 95% CI 1.24–4.23, *P* = 0.008) scores were significantly associated with increased risk of incident ESKD compared to the lower risk score. However, the associations were attenuated after adjustment for cardiometabolic risk factors and baseline renal function (Fig. 4).

Exploratory analysis suggested that the association between plasma ceramide and RDKF was generally consistent across CKD status (eGFR <60 vs. ≥60 ml/min/1.73 m^2^) but statistically significant only in patients without CKD at baseline (Supplemental Table S7). Similar finding was observed across albuminuria category (uACR <30 mg/g, 30–299 mg/g, and ≥300 mg/g) with statistical significance observed mostly in patients with normal albuminuria (Supplemental Table S8). The association between ceramide ratios and ESKD did not differ by CKD status (Supplemental Table S9). In contrast, plasma Cer18:0 and Cer24:0 were negatively associated with risk of ESKD in patients with CKD at baseline (Supplemental Table S9). The association between plasma ceramide ratio, risk score, and ESKD was mostly observed in patients with normal albuminuria (Supplemental Table S10). Interestingly, plasma ceramide ratios and risk score was positively associated with RDKF in patients without CKD and albuminuria at baseline (N = 900) (Supplemental Table S11).

### Additive value of ceramide for prediction of RDKF and ESKD above traditional risk factors

The traditional cardiometabolic risk factors such as age, sex, ethnicity, CVD history, smoking status, BMI, diabetes duration, HbA1c, MAP, lipids level, baseline kidney function (eGFR and uACR), and RAS antagonist usage demonstrated a high discrimination for risk of ESKD (Harrell C-index 0.886; 95% CI 0.852–0.919) in our study. Adding Cer24:1/Cer24:0 ratio significantly improved risk discrimination for ESKD (Harrell C-index 0.895, 95% CI 0.865–0.924, difference 0.01; *P* = 0.022) (Table 3). Similarly, the cardiometabolic risk factors also showed high discrimination for RDKF in our cohort (Harrell C-index 0.811; 95% CI 0.775–0.846). Adding ceramide ratios did not improve the risk prediction for RDKF significantly) (Supplemental Table S12). Similar observations were made when stratified by CKD and albuminuria status at baseline (Supplemental Tables S13 and S14).

**Table 3:** Additive value of ceramide for predicting RDKF and ESKD above traditional risk factors

Model	Variables in Model	ESKD		RDKF	
		AUC (95% CI)	*P*	AUC (95% CI)	*P*
0	Clinical variables	88.6 (85.2–91.9)	-	81.1 (77.5–84.6)	-
1	Clinical variables + Cer24:0	88.6 (85.3–91.9)	0.437	81.4 (77.9–84.9)	0.348
2	Clinical variables + Cer16:0/Cer24:0	89.2 (86.1–92.3)	0.091	81.5 (78.2–84.9)	0.279
**3**	**Clinical variables + Cer24:1/Cer24:0**	**89.5 (86.5–92.4)**	**0.022**	81.9 (78.6–85.3)	0.071
4	Clinical variables + Ceramide score	88.7 (85.4–92.0)	0.101	81.4 (77.9–84.9)	0.146

Clinical variables in model 0 include age, sex, ethnicity, CVD history, smoking status, BMI, diabetes duration, HbA1c, mean arterial pressure, lipids level, baseline kidney function (eGFR and uACR), and RAS antagonist usage. Variables in bold represent *P* value <0.05 compared to the base model 0 (clinical variables). *P* value indicates that the addition of ceramide variable improved the AUC of model 1 (clinical variables + Cer24:0), 2 (clinical variables + Cer16:0/Cer24:0), 3 (clinical variables + Cer24:1/Cer24:0), or 4 (clinical variables + ceramide score), compared to the base model 0 (clinical variables).

Cer, ceramide; eGFR, estimated glomerular filtration function; ESKD, end-stage kidney disease; HbA1c, glycated hemoglobin; RDKF, rapid decline in kidney function; RAS, renin-angiotensin system; uACR, urine albumin-to-creatine ratio.

## Discussion

In this prospective cohort of multiethnic South East Asians with T2D, we demonstrated that specific ceramide concentrations, their ratios, and CERT score conferred a risk of progressive kidney disease beyond traditional cardiometabolic risk factors and baseline renal function (eGFR and albuminuria). Specifically, *1)* a lower level of Cer24:0, a higher level of all three ceramide ratios investigated, and a high-risk ceramide score are associated with an increased likelihood of an early RDKF, assessed as a decline in eGFR of 5 ml/min/1.73 m^2^/year or greater; *2)* a higher level of the ratio Cer16:0/Cer24:0 and Cer24:1/Cer24:0 are associated with increased risk of incident ESKD; *3)* adding Cer24:1/Cer24:0 to clinical risk factors might moderately improve risk discrimination for incident ESKD. Our findings demonstrate that in patients with T2D, ceramides may be a potential novel biomarker for rapid kidney function decline defined by eGFR slope and ESKD.

Our study may be the first to report the robust prospective association of ceramides with progressive kidney disease among T2D patients. Our previous cross-sectional study found that Cer16:0 and Cer18:0 levels are significantly higher in T2D patients with CKD compared to those without CKD (23). In addition, Mantovani *et al.* reported the association of elevated plasma Cer16:0, Cer18:0, Cer20:0, Cer22:0, Cer24:0, and Cer24:1 with CKD in a cross-sectional study on 495 participants (24). Similarly, in this study, we observed that the plasma Cer16:0 and Cer24:1 were significantly higher in T2D patients before experiencing RDKF and ESKD, while Cer18:0 was higher in T2D patients before experiencing RDKF. In the mouse model of DKD, plasma levels Cer16:0 and Cer18:0 positively correlated with uACR but not Cer24:0 and Cer24:1 (21). Here, we observed positive correlation of plasma Cer16:0, Cer18:0, Cer24:0, and Cer24:1 with uACR. Among the four high-risk ceramides measured, only the very long-chain ceramide Cer24:0 was prospectively associated with RDKF. Importantly, an increased level of Cer24:0 was associated with a reduced likelihood of RDKF. This inverse relationship is consistent with previous reports demonstrating the protective role of Cer24:0 with the risk of CVD and mortality (37). Although the underlying mechanism remains unknown, evidence from in vitro and animal studies suggests that Cer24:0 elicits a protective effect against endoplasmic reticulum stress (38) and is also involved in the antiapoptotic mechanism by regulating the formation of membrane channels during the initiation of apoptosis (39, 40). Kidney cell death and tissue fibrosis are common endpoints in most CKD. In this regard, a study by Eckes *et al.* showed that renal fibrosis is associated with lower levels of Cer24:0 in human and mouse fibrotic kidney (41).

Recent studies have shown that ceramide ratios improve risk stratification compared to single ceramide or traditional lipid biomarkers, especially for cardiovascular events (16, 37, 42). In this study, we observed that ratios Cer18:0/Cer24:0, Cer16:0/Cer24:0, and Cer24:1/Cer24:0 were associated with RDKF, with increasing effect size. Specifically, the ratios Cer16:0/Cer24:0 and Cer24:1/Cer24:0 were associated with a 3.5- to 4-fold increase in the likelihood of RDKF and a 3- to 4.5-fold increased risk of incident ESKD. Among the ceramide ratios investigated in the current study, only Cer24:1/Cer24:0 correlated with age, systolic blood pressure, eGFR, and urine albuminuria. Importantly, elevated Cer24:1/Cer24:0 conferred risk of incident ESKD independent of cardio-renal risk factors.

Except for one double-bond, the Cer24:1 and Cer24:0 are structurally identical. The ceramide synthases 2 (CERS2) which is involved in the biosynthesis of very long-chain ceramides, such as Cer24:0 and Cer24:1, is predominantly expressed in liver and kidney (43, 44). Elevated ratio could be a consequence of an increased level of Cer24:1 or decreased level of Cer24:0. Given that increased level of Cer24:0 is associated with a protective effect on kidney function and the CERS2 generates both ceramides, it is unlikely that the elevated ratio is due to deregulation of CERS2 activity. Moreover, only *CERS6* and *CERS3* genes were overexpressed in CKD patients compared to non-CKD (45). Previous studies have demonstrated that fatty acid component alteration between patients with and without CKD. For example, the level of monounsaturated fatty acid increases with decline in kidney function (46). Female patients with CKD had significantly lower levels of Cer16:0 palmitic acid and Cer18:0 stearic acid and increased Cer24:0 lignoceric acid and Cer24:1 nervonic acid levels compared to the control group (47). Therefore, further studies are warranted to understand the factors, including fatty acid availability, in modulating the levels of ceramide ratio and their role in the etiology of progressive DKD.

Currently, ceramide scores are used for risk stratification in patients with known chronic heart disease in primary and secondary prevention settings (17, 18, 27, 28). Similarly, in this study, we found that a high-risk score was independently associated with higher odds of RDKF than those with a lower-risk score. Although a high-risk score was also associated with an increased risk of ESKD, the loss of association after adjustment to uACR and eGFR may be explained by collinearity between ceramide ratios and eGFR. This also suggests that the ceramide score is a better biomarker for early events in progressive kidney disease than in the later stages of T2D. This is consistent with our finding that high risk ceramide score and elevated ceramide ratio were associated with RDKF among patients without CKD and albuminuria at baseline.

The current study has notable strengths. This includes the large sample size, a long duration of follow-up, eGFR trajectories from multiple eGFR readings, and ESRD as an outcome. In addition, biological sample collection predates the advent of renal disease modifying agents such as sodium glucose cotransporter inhibitor and glucagon like peptide 1 receptor agonists.

However, this study has also some limitations. First, due to the nature of the study design, residual confounding is inevitable, and we could not infer causality between ceramide and kidney diseases.

Second, we measured ceramides in the plasma samples, but their source was not determined. Plasma ceramide can be modulated by increased activity of specific CERS, fatty acid availability, diet, inflammatory response, and exercise (29, 48, 49, 50). However, we could not control for these variables in our analysis. Third, ceramide levels were measured at baseline. Hence, we could not test if the changes in ceramide levels over time provide additional evidence to suggest the involvement of ceramide in DKD progression. Similarly, we adjusted for clinical variables at baseline in our analysis. Thus, the contribution of changes in cardio metabolic risk factors could not be accounted for in our analysis. However, adjusting for MAP and HbA1c over follow-up period did not materially change the association between ceramide ratio and RDKF. Fourth, the improvement in C-index although significant was small. This could partly be explained by the significant correlation of ceramide ratio with traditional cardio-renal risk factors. The effect of ceramide ratio could also be diluted with history of standard risk factors. Finally, while absolute differences in ceramide and ceramide ratios were statistically significant, independent studies are needed to validate our findings and assess its clinical utility. However, the CERT score, designed for clinical use with respect to cardiovascular diseases, was associated with RDKF in our study.

In conclusion, we provide evidence that specific ceramides could be susceptibility biomarkers of rapid decline in eGRF and ESKD in patients with T2D. Cer24:0 seems inversely associated with RDKF, while Cer24:1/Cer24:0 appears to be the strongest risk marker for ESKD. More importantly, T2D patients with high-risk ceramide score prior to onset of CKD and albuminuria are susceptible for early RDKF.

## Data Availability

The data supporting this study are available in the article, the supplemental data, or from the corresponding author upon request.

## Supplemental data

This article contains supplemental data.

## Conflict of interest

The authors declare that they have no conflicts of interest with the contents of this article.
