# Supplementary material for: Association of plasma ceramide with decline in kidney function in patients with type 2 diabetes
Source: J Lipid Res. 2024 May 3;65(6):100552. doi: 10.1016/j.jlr.2024.100552 (PMC11176756; doi:10.1016/j.jlr.2024.100552)
Supplement: Supplemental Data [file mmc1.docx]

**Association of plasma ceramide with decline in kidney function in patients with type 2 diabetes**

Resham L GURUNG^1,2^, Yiamunaa M^1^, Wai Kin THAM^3,4^, Sylvia LIU^1^, Huili ZHENG^1^, Janus LEE^1^, Keven ANG^1^, Markus WENK^3,4^, Tavintharan SUBRAMANIAM^5^, Chee Fang SUM^5^ , Federico TORTA^3,4^, Jian-Jun LIU^1^ , Su Chi Lim^1,5,6,7^

^1^Clinical Research Unit, Khoo Teck Puat Hospital, Singapore, Singapore; ^2^Cardiovascular and Metabolic Disorders, Duke-NUS Medical School, Singapore, Singapore; ^3^Precision Medicine Translational Research Programme and Department of Biochemistry, Yong Loo Lin School of Medicine, National University of Singapore, Singapore, Singapore; ^4^SLING, Singapore Lipidomics Incubator, Life Sciences Institute, National University of Singapore, Singapore, Singapore; ^5^Diabetes Centre, Admiralty Medical Centre, Singapore, Singapore; ^6^Saw Swee Hock School of Public Health, Singapore, Singapore; ^7^Lee Kong Chian School of Medicine, Nanyang Technological University, Singapore, Singapore

**Supplementary Tables and Figures**

**Supplementary Table S1:** Association of individual ceramides with rapid decline in kidney function (RDKF)

**Supplementary Table S2:** Association of ceramide ratios with RDKF

**Supplementary Table S3:** Association of ceramide risk score with RDKF

**Supplementary TableS4:** Baseline clinical and biochemical characteristics stratified by end-stage kidney disease (ESKD) status at follow-up

**Supplementary TableS5.** Baseline plasma ceramide levels, ratios and risk group stratified by ESKD status at follow-up

**Supplementary Table S6:** Association of ceramide ratios with ESKD

**Supplementary Table S7**. Association of plasma ceramides, ceramide ratios and ceramide score with RDKF status at follow-up stratified by CKD status

**Supplementary Table S8**. Association of plasma ceramides, ceramide ratios and ceramide score with RDKF status at follow-up stratified by uACR status

**Supplementary Table S95**. Association of plasma ceramides, ceramide ratio and ceramide score with ESKD status at follow-up stratified by CKD status

**Supplementary Table S10**. Association of plasma ceramides, ceramide ratio and ceramide score with ESKD status at follow-up stratified by uACR status

**Supplementary Table S11.** Association of plasma ceramides, ceramide ratio and ceramide score with RDKF status among T2D patients without CKD and albuminuria

**Supplementary Table S12.** Additive value of ceramide for predicting RDKF and ESKD above traditional risk factors, stratified by CKD status

**Supplementary Table S13**. Additive value of ceramide for predicting RDKF and ESKD above traditional risk factors, stratified by uACR status

**Supplementary Table S14.** Additive value of ceramide for predicting RDKF and ESKD above traditional risk factors among those with eGFR > 60ml/min/1.73m^2^ and non-albuminuria

**Supplementary Figure S1:** Study design

**Supplementary Figure S2:** Pearson correlation coefficient between clinical variable

**Supplementary Table S1:** Association of individual ceramides with RDKF

|  | **OR (95% CI)** | **P** | **OR (95% CI)** | **P** | **OR (95% CI)** | **P** | **OR (95% CI)** | **P** |
| --- | --- | --- | --- | --- | --- | --- | --- | --- |
| Cer16:0 | 1.03 (0.87-1.22) | 0.727 | - | - | - | - | - | - |
| Cer18:0 | - | - | 1.01 (0.84-1.21) | 0.920 | - | - | - | - |
| **Cer24:0** | - | - | - | - | **0.71 (0.56-0.90)** | **0.005** | - | - |
| Cer24:1 | - | - | - | - | - | - | 1.07 (0.90-1.27) | 0.472 |
| Age | 0.99 (0.97-1.01) | 0.297 | 0.99 (0.97-1.01) | 0.290 | 0.99 (0.97-1.01) | 0.293 | 0.99 (0.97-1.01) | 0.285 |
| Male | 0.83 (0.57-1.21) | 0.335 | 0.83 (0.57-1.21) | 0.338 | 0.85 (0.58-1.23) | 0.390 | 0.83 (0.57-1.20) | 0.323 |
| Malay ethnicity | **2.52 (1.70-3.75)** | **<0.001** | **2.52 (1.69-3.75)** | **<0.001** | **2.58 (1.73-3.85)** | **<0.001** | **2.53 (1.70-3.77)** | **<0.001** |
| Indian ethnicity | 0.68 (0.42-1.09) | 0.110 | 0.67 (0.42-1.08) | 0.098 | **0.61 (0.38-0.99)** | **0.046** | 0.69 (0.43-1.11) | 0.127 |
| Current smoker | 1.19 (0.67-2.12) | 0.554 | 1.20 (0.68-2.13) | 0.535 | 1.24 (0.70-2.21) | 0.466 | 1.18 (0.66-2.10) | 0.576 |
| Usage of RAS | 1.38 (0.91-2.10) | 0.126 | 1.38 (0.91-2.09) | 0.128 | 1.43 (0.94-2.17) | 0.096 | 1.39 (0.92-2.10) | 0.124 |
| With CVD history | 1.10 (0.60-2.02) | 0.765 | 1.10 (0.60-2.02) | 0.762 | 1.06 (0.58-1.96) | 0.843 | 1.10 (0.60-2.02) | 0.765 |
| BMI | **0.97 (0.93-1.00)** | **0.050** | **0.97 (0.93-1.00)** | **0.050** | **0.96 (0.92-0.99)** | **0.016** | **0.97 (0.93-1.00)** | **0.049** |
| HbA1c | **1.24 (1.09-1.40)** | **0.001** | **1.24 (1.09-1.40)** | **0.001** | **1.25 (1.11-1.42)** | **<0.001** | **1.24 (1.09-1.40)** | **0.001** |
| Duration of diabetes | 1.02 (1.00-1.04) | 0.105 | 1.02 (1.00-1.04) | 0.109 | 1.02 (1.00-1.04) | 0.119 | 1.02 (1.00-1.04) | 0.099 |
| Mean artery pressure | **1.02 (1.00-1.03)** | **0.043** | **1.02 (1.00-1.03)** | **0.043** | 1.02 (1.00-1.03) | 0.059 | **1.02 (1.00-1.03)** | **0.040** |
| LDL-cholesterol | 0.93 (0.74-1.17) | 0.526 | 0.94 (0.76-1.17) | 0.578 | 1.12 (0.87-1.44) | 0.369 | 0.92 (0.73-1.15) | 0.459 |
| Triacylglycerol^*^ | 1.01 (0.71-1.42) | 0.972 | 1.01 (0.71-1.44) | 0.951 | 1.26 (0.84-1.87) | 0.263 | 0.99 (0.70-1.40) | 0.952 |
| Baseline eGFR | 1.01 (1.00-1.02) | 0.119 | 1.01 (1.00-1.02) | 0.125 | 1.01 (1.00-1.02) | 0.096 | 1.01 (1.00-1.02) | 0.112 |
| Urine ACR^*^ | **1.61 (1.46-1.78)** | **<0.001** | **1.61 (1.46-1.78)** | **<0.001** | **1.62 (1.46-1.79)** | **<0.001** | **1.61 (1.45-1.78)** | **<0.001** |

Data were presented as OR (95% CI) per SD increment for individual ceramide. Multivariable model is adjusted for age, sex, ethnicity (Chinese as reference), CVD history (no as reference), smoking status (current smoker versus others), body mass index, diabetes duration, HbA1c, mean arterial pressure, LDL-cholesterol, triglyceride (log-transformed), baseline eGFR and urine ACR (log-transformed) and RAS antagonist usage. Significant observations are bold.

**Supplementary Table S2:** Association of ceramide ratios with RDKF

|  | **OR (95% CI)** | **P** | **OR (95% CI)** | **P** | **OR (95% CI)** | **P** |
| --- | --- | --- | --- | --- | --- | --- |
| Ceramides ratio | - | - | - | - | - | - |
| Cer16:0/24:0 | **3.54 (1.70-7.35)** | **0.001** | - | - | - | - |
| Cer18:0/24:0 | - | - | **1.89 (1.10-3.25)** | **0.022** | - | - |
| Cer24:1/24:0 | - | - | - | - | **4.01 (1.93-8.31)** | **<0.001** |
| Age | 0.99 (0.97-1.01) | 0.376 | 0.99 (0.97-1.01) | 0.268 | 0.99 (0.97-1.01) | 0.256 |
| Male | 0.89 (0.61-1.30) | 0.548 | 0.91 (0.62-1.33) | 0.613 | 0.85 (0.58-1.24) | 0.400 |
| Malay ethnicity | **2.61 (1.75-3.89)** | **<0.001** | **2.64 (1.77-3.93)** | **<0.001** | **2.67 (1.79-4.00)** | **<0.001** |
| Indian ethnicity | 0.70 (0.43-1.13) | 0.140 | 0.67 (0.42-1.08) | 0.100 | 0.73 (0.45-1.18) | 0.200 |
| Current smoker | 1.13 (0.63-2.01) | 0.690 | 1.19 (0.67-2.11) | 0.564 | 1.11 (0.62-2.00) | 0.720 |
| Usage of RAS | 1.47 (0.97-2.24) | 0.072 | 1.42 (0.93-2.16) | 0.101 | 1.48 (0.97-2.26) | 0.067 |
| With CVD history | 1.02 (0.55-1.88) | 0.963 | 1.01 (0.55-1.88) | 0.964 | 1.03 (0.56-1.91) | 0.925 |
| BMI | **0.96 (0.92-0.99)** | **0.016** | **0.96 (0.92-0.99)** | **0.017** | **0.96 (0.92-0.99)** | **0.014** |
| HbA1c | **1.25 (1.10-1.42)** | **<0.001** | **1.24 (1.10-1.41)** | **0.001** | **1.25 (1.11-1.42)** | **<0.001** |
| Duration of diabetes | 1.02 (1.00-1.04) | 0.079 | 1.02 (1.00-1.04) | 0.070 | 1.02 (1.00-1.04) | 0.066 |
| Mean artery pressure | 1.02 (1.00-1.03) | 0.064 | **1.02 (1.00-1.03)** | **0.045** | **1.02 (1.00-1.03)** | **0.046** |
| LDL-cholesterol | 0.99 (0.80-1.22) | 0.894 | 0.98 (0.79-1.22) | 0.878 | 1.00 (0.81-1.24) | 0.987 |
| Triacylglycerol* | 1.09 (0.77-1.54) | 0.625 | 0.99 (0.70-1.41) | 0.971 | 1.02 (0.72-1.44) | 0.910 |
| Baseline eGFR | 1.01 (1.00-1.02) | 0.066 | 1.01 (1.00-1.02) | 0.143 | 1.01 (1.00-1.02) | 0.056 |
| Urine ACR* | **1.61 (1.45-1.78)** | **<0.001** | **1.61 (1.46-1.78)** | **<0.001** | **1.56 (1.44-1.76)** | **<0.001** |

Data were presented as OR (95% CI) per unit increase in natural log transformed ceramide ratio. Multivariable model is adjusted for age, sex, ethnicity (Chinese as reference), CVD history (no as reference), smoking status (current smoker versus others), body mass index, diabetes duration, HbA1c, mean arterial pressure, LDL-cholesterol, triglyceride (log-transformed), baseline eGFR and urine ACR (log-transformed) and RAS antagonist usage. Significant observations are bold.

**Supplementary Table S3:** Association of ceramide risk score with RDKF

|  | **OR (95% CI)** | **P value** |
| --- | --- | --- |
| **Ceramide risk score** |  |  |
| Lower risk (0-2) | reference |  |
| Moderate risk (3-6) | 1.31 (0.85-2.02) | 0.217 |
| Increased risk (7-9) | 1.40 (0.84-2.35) | 0.202 |
| High risk (10-12) | **2.28 (1.26-4.13)** | **0.007** |
| Age | 0.99 (0.97-1.01) | 0.254 |
| Male | 0.88 (0.60-1.28) | 0.505 |
| Malay ethnicity | **2.63 (1.76-3.93)** | **<0.001** |
| Indian ethnicity | 0.73 (0.45-1.18) | 0.197 |
| Current smoker | 1.11 (0.62-1.99) | 0.717 |
| Usage of RAS | 1.45 (0.95-2.20) | 0.085 |
| With CVD history | 0.99 (0.54-1.84) | 0.985 |
| BMI | **0.96 (0.93-0.99)** | **0.022** |
| HbA1c | **1.25 (1.10-1.41)** | **0.001** |
| Duration of diabetes | 1.02 (1.00-1.04) | 0.073 |
| Mean artery pressure | 1.02 (1.00-1.03) | 0.053 |
| LDL-cholesterol | 0.92 (0.74-1.14) | 0.430 |
| Triacylglycerol**^*^** | 0.94 (0.66-1.34) | 0.741 |
| Baseline eGFR | 1.01 (1.00-1.02) | 0.125 |
| Urine ACR^*^ | **1.60 (1.45-1.77)** | **<0.001** |

Triacylglycerol, urine ACR have been log-transformed in the analyses. Low risk score (0-2) was used as reference group for ceramide risk score. Model has been adjusted for age, sex, ethnicity (Chinese as reference), CVD history (Yes with no as reference), smoking status (current smoker versus others as control), body mass index (BMI), diabetes duration, HbA1c, mean arterial pressure, LDL-cholesterol, triglyceride, baseline eGFR, urine ACR (log-transformed) and RAS antagonist usage (Yes versus no as reference). Significant observations are bold.

**Supplementary Table S4**. Baseline clinical and biochemical characteristics stratified by ESKD status at follow-up

|  | **Non-ESKD** | **ESKD** | **P** |
| --- | --- | --- | --- |
|  | (N=1622) | (N=124) |  |
| Index age (years) | 57.2 + 10.6 | 56.2 + 11.6 | 0.289 |
| Male sex (%) | 50.0 | 55.6 | 0.223 |
| **Ethnicity (%)** |  |  |  |
| Chinese | 52.5 | 43.5 | **<0.001** |
| Malay | 19.4 | 41.1 |  |
| Asian Indian | 28.1 | 15.3 |  |
| **Diabetes duration (years)** | **10.9 + 8.8** | **13.5 + 9.4** | **0.001** |
| Active smoker (%) | 8.8 | 10.6 | 0.499 |
| ASCVD history (%) | 8.0 | 7.3 | 0.781 |
| Body mass index (kg/m^2^) | 27.6 + 5.2 | 29.0 + 6.3 | 0.017 |
| **HbA1c (%)** | **7.71 + 1.29** | **8.46 + 1.57** | **<0.001** |
| **Blood pressure (mmHg)** |  |  |  |
| **Systolic** | **139 ± 18** | **150 ± 21** | **<0.001** |
| **Diastolic** | **79 ± 9** | **82 ± 10** | **0.003** |
| **Mean arterial pressure** | **99.0 + 10.4** | **104.4 + 11.9** | **<0.001** |
| Lipid profile (mM) |  |  |  |
| Total cholesterol | 4.39 + 0.99 | 4.55 + 1.08 | 0.086 |
| **HDL cholesterol** | **1.31 + 0.36** | **1.24 + 0.37** | **0.041** |
| LDL cholesterol | 2.73 + 0.80 | 2.84 + 0.90 | 0.150 |
| **Triacylglycerol (IQR)** | **1.37 (1.02-1.89)** | **1.68 (1.19-2.38)** | **<0.001** |
| Baseline renal function |  |  |  |
| **eGFR (ml/min/1.73m^2^)** | **90.2 + 21.9** | **66.9 + 27.8** | **<0.001** |
| **urine ACR (µg/mg)** | **19 (6-66)** | **515 (102-1837)** | **<0.001** |
| Medication usage (%) |  |  |  |
| **RAS antagonist** | **59.0** | **82.9** | **<0.001** |
| **Insulin** | **25.5** | **53.7** | **<0.001** |
| Statin | 80.2 | 85.4 | 0.166 |

Data were presented as mean ± SD, median (interquartile range, IQR) or percentages. Between-group differences were compared by student t test, Mann-Whitney U test or X^2^ test where appropriate.

Abbreviations; ASCVD, atherosclerotic cardiovascular disease; eGFR, estimated glomerular filtration function; ACR, albumin-to-creatinine ratio. Variables differed significantly between groups have been highlighted in bold font.

**Supplementary Table S5.** Baseline plasma ceramide levels stratified by ESKD status at follow-up

|  | **All participants** | **Non-ESKD** | **ESKD** | **P** |
| --- | --- | --- | --- | --- |
| ***Individual ceramide*** |  |  |  |  |
| Cer16:0 | 0.236 (0.200-0.279) | 0.234 (0.198-0.277) | 0.250 (0.218-0.300) | **0.001** |
| Cer18:0 | 0.107 (0.084-0.134) | 0.107 (0.084-0.134) | 0.111 (0.085-0.140) | 0.293 |
| Cer24:0 | 3.500 (2.887-4.198) | 3.500 (2.888-4.185) | 3.482 (2.792-4.354) | 0.389 |
| Cer24:1 | 1.044 (0.853-1.264) | 1.034 (0.847-1.248) | 1.177 (0.928-1.426) | **<0.001** |
| ***Ratios with Cer24:0*** |  |  |  |  |
| Cer16:0/Cer24:0 | 0.067 (0.059-0.078) | 0.067 (0.059-0.078) | 0.070 (0.059-0.085) | **0.010** |
| Cer18:0/Cer24:0 | 0.031 (0.025-0.038) | 0.031 (0.025-0.038) | 0.031 (0.024-0.037) | 0.448 |
| Cer24:1/Cer24:0 | 0.302 (0.256-0.351) | 0.300 (0.255-0.349) | 0.320 (0.291-0.377) | **<0.001** |
|  |  |  |  |  |
| **Ceramide risk score** |  |  |  | **0.016** |
| Lower risk (0-2) | 593 (34.0) | 565 (34.9) | 28 (22.6) |  |
| Moderate risk (3-6) | 670 (38.4) | 621 (38.3) | 49 (39.5) |  |
| Increased risk (7-9) | 320 (18.3) | 289 (17.8) | 31 (25.0) |  |
| High risk (10-12) | 163 (9.3) | 146 (9.0) | 16 (12.9) |  |

Data are presented as median [IQR] for individual ceramide (μM) and ratios, and proportion (n, %) for ceramide risk score. Abbreviation: Cer, ceramide; ESKD, end stage kidney disease. Significant observations are bold.

|  | **HR (95% CI)** | **P value** | **HR (95% CI)** | **P value** | **HR (95% CI)** | **P value** |
| --- | --- | --- | --- | --- | --- | --- |
| Ceramides ratio* | - | - | - | - | - | - |
| Cer16:0/24:0 | **3.10 (1.44-6.64)** | **0.004** | - | - | - | - |
| Cer18:0/24:0 | - | - | 1.18 (0.64-2.19) | 0.594 | - | - |
| Cer24:1/24:0 | - | - | - | - | **4.66 (1.93-11.24)** | **0.0006** |
| Age | **0.96 (0.93-0.98)** | **<0.0001** | **0.95 (0.93-0.97)** | **<0.0001** | **0.95 (0.93-0.97)** | **<0.0001** |
| Male | 0.94 (0.63-1.40) | 0.744 | 0.90 (0.60-1.38) | 0.640 | 0.94 (0.63-1.40) | 0.752 |
| Malay ethnicity | **1.79 (1.17-2.75)** | **0.008** | **1.80 (1.17-2.77)** | **0.008** | **1.83 (1.19-2.82)** | **0.006** |
| Indian ethnicity | 0.81 (0.47-1.40) | 0.451 | 0.81 (0.47-1.40) | 0.453 | 0.84 (0.48-1.44) | 0.519 |
| Current smoker | 0.59 (0.31-1.13) | 0.114 | 0.63 (0.33-1.20) | 0.160 | 0.56 (0.29-1.07) | 0.078 |
| Usage of RAS | 0.92 (0.45-1.86) | 0.808 | 0.96 (0.47-1.96) | 0.915 | 0.95 (0.47-1.93) | 0.888 |
| With CVD history | 1.11 (0.67-1.83) | 0.694 | 1.06 (0.64-1.75) | 0.824 | 1.11 (0.67-1.83) | 0.696 |
| BMI | 0.98 (0.94-1.02) | 0.371 | 0.99 (0.95-1.03) | 0.634 | 0.98 (0.94-1.02) | 0.308 |
| HbA1c | **1.32 (1.16-1.49)** | **<0.0001** | **1.29 (1.14-1.46)** | **<0.0001** | **1.31 (1.16-1.49)** | **<0.0001** |
| Duration of diabetes | 0.99 (0.97-1.02) | 0.544 | 0.99 (0.97-1.02) | 0.613 | 0.99 (0.97-1.01) | 0.450 |
| Mean artery pressure | 1.00 (0.98-1.02) | 0.983 | 1.00 (0.98-1.02) | 0.810 | 1.00 (0.98-1.02) | 0.953 |
| LDL-cholesterol | 0.90 (0.71-1.14) | 0.381 | 0.85 (0.67-1.08) | 0.175 | 0.90 (0.71-1.13) | 0.365 |
| Triacylglycerol* | 1.00 (0.69-1.44) | 0.989 | 0.96 (0.67-1.38) | 0.823 | 0.98 (0.68-1.40) | 0.901 |
| Baseline eGFR | **0.96 (0.96-0.97)** | **<0.0001** | **0.96 (0.95-0.97)** | **<0.0001** | **0.96 (0.96-0.97)** | **<0.0001** |
| Urine ACR* | **1.86 (1.66-2.09)** | **<0.0001** | **1.87 (1.67-2.09)** | **<0.0001** | **1.86 (1.66-2.08)** | **<0.0001** |

**Supplementary Table S6:** Association of ceramide ratios with ESKD

Data were presented as HR (95% CI) per SD for per natural log transformed for ceramide ratio. Multivariable model is adjusted for age, sex, ethnicity (Chinese as reference), CVD history (no as reference), smoking status (current smoker versus others), body mass index, diabetes duration, HbA1c, mean arterial pressure, LDL-cholesterol, triglyceride, baseline eGFR and urine ACR (log-transformed) and RAS antagonist usage. Significant observations are bold.

**Supplementary Table S7.** Association of plasma ceramides, ceramide ratios and ceramide score with RDKF status at follow-up stratified by CKD status

| **-** | **Unadjusted** | | | | **Multivariable** | | | |
| --- | --- | --- | --- | --- | --- | --- | --- | --- |
| **CKD status**  **(N, case/control)** | **eGFR> 60ml/min/1.73m^2^ (157/1330)** | | **eGFR<60ml/min/1.73m^2^ (40/218)** | | **eGFR> 60ml/min/1.73m^2^ (153/1302)** | | **eGFR<60ml/min/1.73m^2^ (40/210)** | |
|  | **OR (95% CI)** | **P** | **OR (95% CI)** | **P** | **OR (95% CI)** | **P** | **OR (95% CI)** | **P** |
| ***Individual Ceramides*** | - | - | - | - | - | - | - | - |
| Cer16:0 | 1.15 (0.97-1.37) | 0.111 | **1.62 (1.09-2.39)** | **0.016** | 1.00 (0.83-1.21) | 0.989 | 1.32 (0.71-2.44) | 0.379 |
| Cer18:0 | 1.14 (0.96-1.36) | 0.125 | 1.06 (0.73-1.54) | 0.756 | 1.03 (0.80-1.98) | 0.801 | 0.93 (0.53-1.63) | 0.793 |
| Cer24:0 | 0.99 (0.84-1.17) | 0.927 | 1.01 (0.71-1.42) | 0.965 | **0.72 (0.56-0.94)** | **0.016** | 0.61 (0.33-1.11) | 0.105 |
| Cer24:1 | **1.27 (1.07-1.50)** | **0.006** | 1.34 (0.91-1.99) | 0.139 | 1.09 (0.91-1.30) | 0.367 | 0.98 (0.54-1.79) | 0.954 |
| ***Ceramide Ratios*** | - | - | - | - | - | - | - | - |
| Cer16:0/Cer24:0 | **2.09 (1.05-4.15)** | **0.036** | **4.36 (1.25-15.18)** | **0.021** | **3.19 (1.38-7.36)** | **0.007** | 5.08 (1.00-25.88) | 0.050 |
| Cer18:0/Cer24:0 | 1.58 (0.97-2.58) | 0.066 | 1.15 (0.43-3.06) | 0.777 | **1.99 (1.08-3.64)** | **0.027** | 1.83 (0.47-7.12) | 0.383 |
| Cer24:1/Cer24:0 | **3.38 (1.74-6.59)** | **<0.0001** | 2.84 (0.76-10.52) | 0.119 | **4.68 (2.01-10.62)** | **<0.0001** | 3.19 (0.57-17.31) | 0.180 |
| ***Ceramide risk scores*** | - | - | - | - | - | - | - | - |
| Lower risk (0-2) | ref | ref | ref | ref | ref | ref | ref | ref |
| Moderate risk (3-6) | 1.47 (0.97-2.23) | 0.069 | 2.04 (0.76-5.52) | 0.160 | 1.26 (0.79-2.02) | 0.329 | 1.49 (0.46-4.91) | 0.508 |
| Increased risk (7-9) | **1.72 (1.06-2.82)** | **0.030** | 1.99 (0.68-5.84) | 0.211 | 1.48 (0.84-2.63) | 0.179 | 1.25 (0.35-4.54) | 0.733 |
| High risk (10-12) | **2.56 (1.47-4.45)** | **0.001** | **3.59 (1.13-11.48)** | **0.031** | **2.26 (1.16-4.38)** | **0.016** | 3.41 (0.72-16.18) | 0.122 |

Data were presented as OR (95% CI) per SD for individual ceramide or per natural log transformed for ceramide ratio. Multivariable models are adjusted for age, sex, ethnicity (Chinese as reference), CVD history (no as reference), smoking status (current smoker versus others), body mass index, diabetes duration, HbA1c, mean arterial pressure, LDL-cholesterol, triglyceride, baseline eGFR and urine ACR (log-transformed) and RAS antagonist usage. Significant observations are bold.

**Supplementary Table S8.** Association of plasma ceramides, ceramide ratio and ceramide score with RDKF status at follow-up stratified by uACR status

| **-** | **Unadjusted** | | | | | | **Multivariable** | | | | | |
| --- | --- | --- | --- | --- | --- | --- | --- | --- | --- | --- | --- | --- |
| **uACR status (N, case/control)** | **Non-albuminuria (uACR<30mg/g)**  **(43/941)** | | **Mircoalbuminuria (uACR=30-299mg/g) (63/460)** | | **Macroalbuminuria (uACR>300)**  **(90/140)** | | **Non-albuminuria (uACR<30mg/g)**  **(42/925)** | | **Mircoalbuminuria (uACR=30-299mg/g) (63/448)** | | **Macroalbuminuria (uACR>300)**  **(88/139)** | |
|  | **OR**  **(95% CI)** | **P** | **OR**  **(95% CI)** | **P** | **OR**  **(95% CI)** | **P** | **OR**  **(95% CI)** | **P** | **OR**  **(95% CI)** | **P** | **OR**  **(95% CI)** | **P** |
| **Individual Ceramides** | - | - | - | - | - | - | - | - | - | - | - | - |
| Cer16:0 | 1.36  (0.99-1.88) | 0.057 | 1.20  (0.91-1.57) | 0.193 | 0.99  (0.82-1.19) | 0.878 | 1.20  (0.77-1.87) | 0.431 | 0.98  (0.69-1.40) | 0.911 | 1.03  (0.77-1.37) | 0.854 |
| Cer18:0 | **1.54  (1.12-2.13)** | **0.008** | 0.94  (0.71-1.24) | 0.646 | 0.91  (0.73-1.15) | 0.435 | **1.58  (1.07-2.33)** | **0.022** | **0.69  (0.48-0.99)** | **0.042** | 1.07  (0.79-1.43) | 0.670 |
| Cer24:0 | 1.00  (0.73-1.37) | 0.982 | 0.92  (0.70-1.21) | 0.533 | 0.92  (0.75-1.14) | 0.465 | **0.65  (0.43-0.99)** | **0.043** | 0.69  (0.47-1.01) | 0.056 | 0.69  (0.42-1.12) | 0.130 |
| Cer24:1 | **1.45  (0.16-1.99)** | **0.020** | 1.19  (0.91-1.56) | 0.213 | 0.93  (0.75-1.16) | 0.516 | 1.42  (0.93-2.15) | 0.104 | 1.07  (0.75-1.54) | 0.704 | 0.89  (0.59-1.35) | 0.592 |
| **Ceramide Ratios** | - | - | - | - | - | - | - | - | - | - | - | - |
| Cer16:0/Cer24:0 | **3.57  (1.13-11.36)** | **0.031** | **3.52  (1.18-10.47)** | **0.024** | 1.73  (0.56-5.33) | 0.342 | **5.64  (1.59-19.99)** | **0.007** | 3.05  (0.90-10.37) | 0.074 | 4.55  (1.00-20.75) | 0.051 |
| Cer18:0/Cer24:0 | **3.87  (1.56-9.57)** | **0.003** | 1.01  (0.46-2.22) | 0.972 | 0.90  (0.40-2.01) | 0.794 | **5.31  (2.04-13.85)** | **0.001** | 0.75  (0.30-1.90) | 0.541 | 2.81  (0.91-8.65) | 0.072 |
| Cer24:1/Cer24:0 | **5.01  (1.59-15.76)** | **0.006** | **3.40  (1.17-9.87)** | **0.025** | 1.07  (0.34-3.41) | 0.904 | **8.49  (2.42-29.81)** | **0.001** | **4.29  (1.23-15.00)** | **0.022** | 2.10  (0.48-9.23) | 0.328 |
| **Ceramide risk scores** | - | - | - | - | - | - | - | - | - | - | - | - |
| Lower risk (0-2) | ref | ref | ref | ref | ref | ref | ref | ref | ref | ref | ref | ref |
| Moderate risk (3-6) | 1.53  (0.70-3.34) | 0.286 | 2.25  (1.104.62) | 0.027 | 0.81  (0.41-1.61) | 0.547 | 1.42  (0.63-3.20) | 0.395 | 2.22  (1.00-4.92) | 0.051 | 0.77  (0.34-1.75) | 0.526 |
| Increased risk (7-9) | 1.66  (0.66-4.21) | 0.285 | 1.73  (0.72-4.16) | 0.221 | 0.97  (0.46-2.06) | 0.972 | 1.84  (0.68-4.97) | 0.228 | 1.68  (0.63-4.45) | 0.298 | 1.21  (0.48-3.06) | 0.684 |
| High risk (10-12) | **3.57  (1.39-9.17)** | **0.008** | **3.17  (1.22-8.19)** | **0.017** | 1.13  (0.48-2.71) | 0.777 | **3.79  (1.30-11.00)** | **0.014** | 2.58  (0.86-7.73) | 0.089 | 1.48  (0.79-4.49) | 0.488 |

Data were presented as OR (95% CI) per SD for individual ceramide or per natural log transformed for ceramide ratio. Multivariable models are adjusted for age, sex, ethnicity (Chinese as reference), CVD history (no as reference), smoking status (current smoker versus others), body mass index, diabetes duration, HbA1c, mean arterial pressure, LDL-cholesterol, triglyceride, baseline eGFR and urine ACR (log-transformed) and RAS antagonist usage. Significant observations are bold.

**Supplementary Table S9.** Association of plasma ceramides, ceramide ratios and ceramide score with ESKD status at follow-up stratified by CKD status

| **-** | **Unadjusted** | | | | **Multivariable** | | | |
| --- | --- | --- | --- | --- | --- | --- | --- | --- |
| **CKD status**  **(N, case/control)** | **eGFR> 60ml/min/1.73m^2^ (60/1427)** | | **eGFR<60ml/min/1.73m^2^ (64/193)** | | **eGFR> 60ml/min/1.73m^2^ (59/1396)** | | **eGFR<60ml/min/1.73m2 (63/187)** | |
| - | **HR (95% CI)** | **P** | **HR (95% CI)** | **P** | **HR (95% CI)** | **P** | **HR (95% CI)** | **P** |
| **Individual Ceramides** | - | - | - | - | - | - | - | - |
| Cer16:0 | **1.30 (1.00-1.68)** | **0.047** | **1.43 (1.07-1.91)** | **0.015** | 1.30 (0.65-2.62) | 0.316 | 0.94 (0.58-1.52) | 0.804 |
| Cer18:0 | 1.18 (0.91-1.54) | 0.218 | 0.99 (0.76-1.31) | 0.965 | 1.10 (0.88-1.37) | 0.416 | **0.70 (0.49-1.00)** | **0.048** |
| Cer24:0 | 1.06 (0.82-1.38) | 0.641 | 1.03 (0.80-1.33) | 0.818 | 1.02 (0.85-1.23) | 0.827 | **0.58 (0.38-0.86)** | **0.007** |
| Cer24:1 | **1.39 (1.09-1.79)** | **0.009** | **1.49 (1.11-1.99)** | **0.008** | 1.19 (0.91-1.55) | 0.215 | 0.97 (0.65-1.43) | 0.862 |
| **Ceramide Ratios** | - | - | - | - | - | - | - | - |
| Cer16:0/Cer24:0 | 2.46 (0.93-6.52) | 0.071 | **2.70 (1.08-6.75)** | **0.033** | **3.24 (1.04-10.06)** | **0.042** | **3.44 (1.13-10.42)** | **0.029** |
| Cer18:0/Cer24:0 | 1.46 (0.69-3.07) | 0.323 | 0.92 (0.45-1.87) | 0.812 | 1.79 (0.74-4.37) | 0.198 | 0.96 (0.41-2.26) | 0.959 |
| Cer24:1/Cer24:0 | **3.78 (1.43-9.96)** | **0.007** | **3.76 (1.41-10.02)** | **0.008** | **5.34 (1.53-18.67)** | **0.009** | **4.17 (1.18-14.74)** | **0.027** |
| **Ceramide risk scores** | - | - | - | - | - | - | - | - |
| Lower risk (0-2) | ref | ref | ref | ref | ref | ref | ref | ref |
| Moderate risk (3-6) | 0.64 (0.25-1.63) | 0.346 | **0.42 (0.18-0.97)** | **0.041** | 1.32 (0.70-2.51) | 0.396 | 1.14 (0.54-2.41) | 0.725 |
| Increased risk (7-9) | 0.97 (0.40-2.36) | 0.952 | 0.60 (0.29-1.26) | 0.177 | 1.47 (0.65-3.34) | 0.354 | 1.24 (0.56-2.78) | 0.596 |
| High risk (10-12) | 0.99 (0.37-2.63) | 0.977 | 0.93 (0.43-2.01) | 0.855 | 1.33 (0.48-3.63) | 0.585 | 1.50 (0.54-4.18) | 0.434 |

Data were presented as HR (95% CI) per SD for individual ceramide or per natural log transformed for ceramide ratio. Multivariable models are adjusted for age, sex, ethnicity (Chinese as reference), CVD history (no as reference), smoking status (current smoker versus others), body mass index, diabetes duration, HbA1c, mean arterial pressure, LDL-cholesterol, triglyceride, baseline eGFR and urine ACR (log-transformed) and RAS antagonist usage. Significant observations are bold.

**Supplementary Table S10.** Association of plasma ceramides, ceramide ratio and ceramide score with ESKD status at follow-up stratified by uACR status

| **-** | **Unadjusted** | | | | | | **Multivariable** | | | | | |
| --- | --- | --- | --- | --- | --- | --- | --- | --- | --- | --- | --- | --- |
| **uACR status (N, case/control)** | **Non-albuminuria (uACR<30mg/g)**  **(14/961)** | | **Mircoalbuminuria**  **(uACR=29-299mg/g) (37/486)** | | **Macroalbuminuria**  **(uACR>300) (73/157)** | | **Non-albuminuria (uACR<30mg/g)**  **(13/946)** | | **Mircoalbuminuria**  **(uACR=29-299mg/g) (37/474)** | | **Macroalbuminuria**  **(uACR>300)**  **(72/155)** | |
| **-** | **OR**  **(95% CI)** | **P** | **OR**  **(95% CI)** | **P** | **OR**  **(95% CI)** | **P** | **OR**  **(95% CI)** | **P** | **OR**  **(95% CI)** | **P** | **OR**  **(95% CI)** | **P** |
| **Individual Ceramides** | - | - | - | - | - | - | - | - | - | - | - | - |
| Cer16:0 | 1.60  (0.95-2.71) | 0.078 | 1.03  (0.73-1.44) | 0.878 | **1.46  (1.10-1.94)** | **0.008** | 1.62  (0.79-3.34) | 0.189 | 0.85  (0.54-1.32) | 0.460 | 1.09  (0.85-1.39) | 0.510 |
| Cer18:0 | 1.49  (0.85-2.58) | 0.162 | 0.84  (0.60-1.19) | 0.329 | 1.01  (0.84-1.21) | 0.924 | 1.59  (0.80-3.16) | 0.190 | 0.70  (0.46-1.08) | 0.104 | 1.01  (0.82-1.26) | 0.899 |
| Cer24:0 | 0.84  (0.50-1.42) | 0.518 | 0.75  (0.53-1.05) | 0.095 | 1.18  (0.93-1.51) | 0.176 | 0.60  (0.29-1.22) | 0.156 | 0.68  (0.42-1.09) | 0.111 | 0.96  (0.79-1.15) | 0.631 |
| Cer24:1 | **1.87  (1.11-3.15)** | **0.018** | 1.12  (0.80-1.55) | 0.522 | **1.36  (1.04-1.77)** | **0.024** | 2.02  (0.97-4.23) | 0.062 | 1.06  (0.64-1.76) | 0.823 | 1.10  (0.84-1.43) | 0.429 |
| **Ceramide Ratios** | - | - | - | - | - | - | - | - | - | - | - | - |
| Cer16:0/Cer24:0 | **7.37  (2.04-26.65)** | **0.002** | **4.14  (1.25-13.76)** | **0.020** | 2.24  (0.82-6.13) | 0.116 | **10.67  (1.63-70.09)** | **0.014** | 1.65  (0.41-6.68) | 0.484 | **3.15  (1.09-9.12)** | **0.034** |
| Cer18:0/Cer24:0 | **5.10  (1.13-23.02)** | **0.034** | 1.19  (0.46-3.08) | 0.717 | 0.60  (0.31-1.20) | 0.148 | **6.54  (1.25-34.15)** | **0.026** | 0.76 (0.26-2.17) | 0.603 | 1.46  (0.60-3.56) | 0.403 |
| Cer24:1/Cer24:0 | **17.41  (3.99-75.90)** | **<0.001** | **6.31  (1.77-22.49)** | **0.004** | 2.01  (0.70-5.73) | 0.192 | **21.28  (3.16-143.48)** | **0.002** | 4.52  (0.90-22.69) | 0.067 | **5.72  (1.43-22.99)** | **0.014** |
| **Ceramide risk scores** | - | - | - | - | - | - | - | - | - | - | - | - |
| Lower risk (0-2) | ref | ref | ref | ref | ref | ref | ref | ref | ref | ref | ref | ref |
| Moderate risk (3-6) | 1.75  (0.42-7.31) | 0.445 | 1.45  (0.62-3.38) | 0.395 | 1.39  (0.76-2.55) | 0.290 | 1.90  (0.44-8.24) | 0.389 | 1.17  (0.47-2.93) | 0.735 | 1.13  (0.58-2.19) | 0.715 |
| Increased risk (7-9) | 2.23  (0.46-11.26) | 0.315 | 1.94 (0.75-5.02) | 0.174 | 1.49  (0.77-2.89) | 0.236 | 1.32  (0.20-8.77) | 0.774 | 1.32 (0.47-3.73) | 0.601 | 1.50  (0.70-3.23) | 0.296 |
| High risk (10-12) | **5.01  (1.01-24.82)** | **0.048** | 1.75  (0.53-5.81) | 0.361 | 1.16  (0.52-2.63) | 0.714 | **4.26  (0.71-25.55)** | **0.113** | 1.11  (0.30-4.20) | 0.873 | 1.09  (0.42-2.86) | 0.861 |

Data were presented as HR (95% CI) per SD for individual ceramide or per natural log transformed for ceramide ratio. Multivariable model is adjusted for age, sex, ethnicity (Chinese as reference), CVD history (no as reference), smoking status (current smoker versus others), body mass index, diabetes duration, HbA1c, mean arterial pressure, LDL-cholesterol, triglyceride, baseline eGFR and urine ACR (log-transformed) and RAS antagonist usage. Significant observations are bold.

**Supplementary Table S11.** Association of plasma ceramides, ceramide ratio and ceramide score with RDKF status among T2D patients without CKD and albuminuria.

| **(N, case/control)** | **Unadjusted**  **(40/860)** | | **Multivariable**  **(39/847)** | |
| --- | --- | --- | --- | --- |
|  | **OR (95% CI)** | **P** | **OR (95% CI)** | **P** |
| ***Individual Ceramides*** | - | - | - | - |
| Cer16:0 | 1.29 (0.93-1.80) | 0.127 | 1.13 (0.71-1.80) | 0.610 |
| Cer18:0 | **1.47 (1.06-2.05)** | **0.021** | 1.48 (0.99-2.21) | 0.054 |
| Cer24:0 | 1.00 (0.72-1.38) | 0.993 | 0.66 (0.43-1.03) | 0.064 |
| Cer24:1 | **1.38 (1.00-1.92)** | **0.050** | 1.34 (0.87-2.06) | 0.181 |
| ***Ceramide Ratios*** | - | - | - | - |
| Cer16:0/Cer24:0 | 3.01 (0.88-10.28) | 0.080 | **4.91 (1.26-19.05)** | **0.022** |
| Cer18:0/Cer24:0 | **3.37 (1.32-8.58)** | **0.011** | **4.76 (1.73-13.07)** | **0.002** |
| Cer24:1/Cer24:0 | **4.17 (1.25-13.88)** | **0.020** | **7.52 (1.97-28.64)** | **0.003** |
| ***Ceramide risk scores*** | - | - | - | - |
| Lower risk (0-2) | ref | ref | ref | ref |
| Moderate risk (3-6) | 1.70 (0.78-3.79) | 0.198 | 1.59 (0.69-3.67) | 0.276 |
| Increased risk (7-9) | 1.83 (0.71-4.73) | 0.212 | 2.13 (0.76-5.93) | 0.150 |
| High risk (10-12) | **3.08 (1.08-8.78)** | **0.035** | **3.26 (1.01-10.50)** | **0.048** |

Data were presented as OR (95% CI) per SD for individual ceramide or per natural log transformed for ceramide ratio. Multivariable model is adjusted for age, sex, ethnicity (Chinese as reference), CVD history (no as reference), smoking status (current smoker versus others), body mass index, diabetes duration, HbA1c, mean arterial pressure, LDL-cholesterol, triglyceride, baseline eGFR and urine ACR (log-transformed) and RAS antagonist usage. Significant observations are bold.

**Supplementary Table S12.** Additive value of ceramide for predicting RDKF and ESKD above traditional risk factors, stratified by CKD status

|  | **ESKD** | | | | **RDKF** | | | |
| --- | --- | --- | --- | --- | --- | --- | --- | --- |
|  | **eGFR> 60ml/min/1.73m^2^** | | **eGFR< 60ml/min/1.73m^2^** | | **eGFR> 60ml/min/1.73m^2^** | | **eGFR< 60ml/min/1.73m^2^** | |
| **Model** | **AUC (95%CI)** | **P** | **AUC (95%CI)** | **P** | **AUC (95%CI)** | **P** | **AUC (95%CI)** | **P** |
| 0 | 86.8 (81.5-92.0) |  | 83.6 (77.9-89.2) |  | 80.8 (76.7-84.8) |  | 85.9 (79.3-92.5) |  |
| 1 | 86.8 (81.5-92.0) | 0.635 | 84.4 (78.9-90.0) | 0.170 | 81.0 (77.1-84.9) | 0.605 | 86.4 (80.0-92.8) | 0.471 |
| 2 | 87.3 (82.3-92.3) | 0.345 | 84.0 (78.6-89.4) | 0.524 | 81.1 (77.2-84.9) | 0.518 | 86.7 (80.7-92.8) | 0.349 |
| 3 | 87.7 (82.8-92.5) | 0.081 | 84.1 (78.9-89.4) | 0.423 | 81.8 (78.0-85.6) | 0.097 | 86.1 (79.8-92.3) | 0.835 |
| 4 | 87.0 (81.8-92.2) | 0.314 | 83.7 (78.1-89.2) | 0.743 | 81.1 (77.1-85.1) | 0.281 | 85.9 (79.4-92.4) | 0.938 |

Clinical variables in model 0 include age, sex, ethnicity, CVD history, smoking status, BMI, diabetes duration, HbA1c, mean arterial pressure, lipids level, baseline kidney function (eGFR and uACR) and RAS antagonist usage. P-value <0.05 indicates that the addition of ceramide improved the AUC of model 1 (clinical variables + Cer24:0), 2 (clinical variables + Cer16:0/Cer24:0), 3 (clinical variables + Cer24:1/Cer24:0) or 4 (clinical variables + ceramide score), compared to the base model 0 (clinical variables).

**Supplementary Table S13**. Additive value of ceramide for predicting RDKF and ESKD above traditional risk factors, stratified by uACR status

|  | **ESKD** | | | | | | **RDKF** | | | | | |
| --- | --- | --- | --- | --- | --- | --- | --- | --- | --- | --- | --- | --- |
|  | **Non-albuminuria**  **(uACR<30mg/g)** | | **Microalbuminuria**  **(uACR=30-299mg/g)** | | **Macro albuminuria**  **(uACR>300mg/g)** | | **Non-albuminuria**  **(uACR<30mg/g)** | | **Microalbuminuria**  **(uACR=30-299mg/g)** | | **Macroalbuminuria**  **(uACR>300mg/g)** | |
| **Model** | **AUC (95%CI)** | **P** | **AUC (95%CI)** | **P** | **AUC (95%CI)** | **P** | **AUC (95%CI)** | **P** | **AUC (95%CI)** | **P** | **AUC (95%CI)** | **P** |
| 0 | 78.3 (66.8-89.8) |  | 82.5 (75.3-89.6) |  | 81.6 (76.6-86.7) |  | 71.7 (63.8-79.5) |  | 74.3 (67.4-81.3) |  | 78.7 (72.6-84.7) |  |
| 1 | 80.0 (67.1-93.0) | 0.601 | 82.8 (75.9-89.6) | 0.777 | 81.8 (76.7-86.8) | 0.316 | 73.6 (66.4-80.9) | 0.411 | 75.2 (68.4-82.0) | 0.402 | 79.3 (73.3-85.2) | 0.403 |
| 2 | 80.4 (67.7-93.1) | 0.510 | 82.6 (75.6-89.7) | 0.664 | 82.6 (77.7-87.4) | 0.124 | 74.4 (67.0-81.9) | 0.304 | 74.6 (67.8-81.4) | 0.690 | 79.7 (73.8-85.7) | 0.319 |
| 3 | 83.3 (73.3-93.3) | 0.060 | 83.7 (77.3-90.1) | 0.126 | 82.5 (77.6-87.4) | 0.239 | 75.3 (67.0-83.7) | 0.247 | 75.4 (68.7-82.2) | 0.360 | 78.7 (72.7-84.7) | 0.919 |
| 4 | 79.3 (67.9-90.6) | 0.515 | 82.6 (75.5-89.6) | 0.691 | 81.9 (76.9-86.9) | 0.374 | 72.4 (64.3-80.5) | 0.654 | 75.7 (69.3-82.1) | 0.181 | 79.4 (73.4-85.4) | 0.318 |

Clinical variables in model 0 include age, sex, ethnicity, CVD history, smoking status, BMI, diabetes duration, HbA1c, mean arterial pressure, lipids level, baseline kidney function (eGFR and uACR) and RAS antagonist usage. P-value <0.05 indicates that the addition of ceramide improved the AUC of model 1 (clinical variables + Cer24:0), 2 (clinical variables + Cer16:0/Cer24:0), 3 (clinical variables + Cer24:1/Cer24:0) or 4 (clinical variables + ceramide score), compared to the base model 0 (clinical variables).

**Supplementary Table S14.** Additive value of ceramide for predicting RDKF and ESKD above traditional risk factors among those with eGFR > 60ml/min/1.73m^2^ and non-albuminuria

|  |  | **ESKD** | | **RDKF** | |
| --- | --- | --- | --- | --- | --- |
| **Model** | **Variables in model** | **AUC (95%CI)** | **P** | **AUC (95%CI)** | **P** |
| 0 | Clinical variables | 80.0 (69.8-90.1) |  | 71.0 (62.6-79.4) |  |
| 1 | Clinical variables + Cer16:0/Cer24:0 | 85.5 (79.4-91.7) | 0.268 | 73.8 (65.7-81.8) | 0.353 |
| 2 | Clinical variables + Cer18:0/Cer24:0 | 85.9 (80.7-91.2) | 0.102 | 74.6 (65.7-83.4) | 0.295 |
| 3 | Clinical variables + Cer24:1/Cer24:0 | 83.5 (77.0-90.0) | 0.350 | 75.9 (68.4-83.4) | 0.093 |
| 4 | Clinical variables + Ceramide score | 82.9 (68.1-97.7) | 0.525 | 71.8 (63.0-80.6) | 0.676 |

Clinical variables in model 0 include age, sex, ethnicity, CVD history, smoking status, BMI, diabetes duration, HbA1c, mean arterial pressure, lipids level, baseline kidney function (eGFR and uACR) and RAS antagonist usage. P-value <0.05 indicates that the addition of ceramide improved the AUC of model 1, 2, 3 or 4, compared to the base model 0.

**Supplementary Figure S1 :** Study design


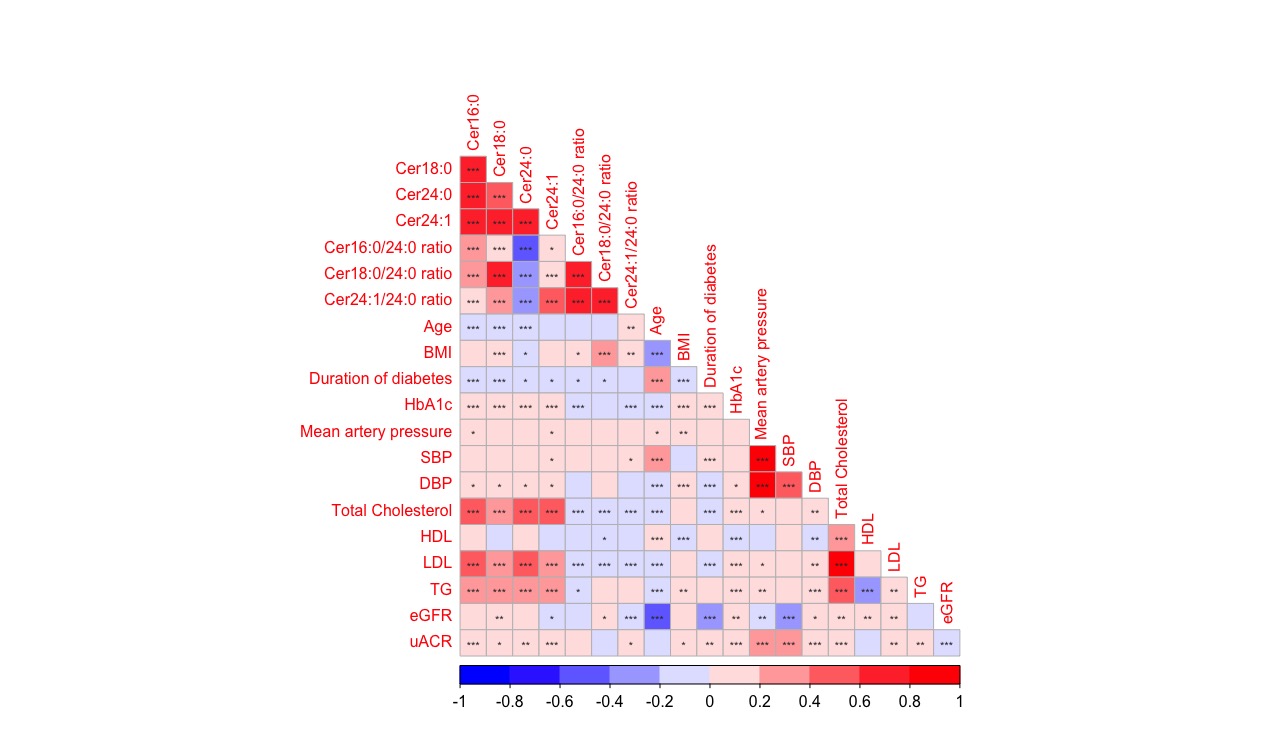


**Supplementary Figure S2. Pearson correlation coefficient between clinical variables.**

Variables significantly correlated are indicated by (*).*P<0.05,**P<0.001,***P<0.0001
